# Identification of Changes in Gene Expression

**DOI:** 10.1002/bimj.70181

**Published:** 2026-09-24

**Authors:** Lucia Ameis, Kathrin Möllenhoff

**Affiliations:** ^1^ Institute of Medical Statistics and Computational Biology (IMSB), Faculty of Medicine University of Cologne Cologne Germany

**Keywords:** bootstrap, gene expression data, model‐based hypothesis testing, preclinical research, time‐response models

## Abstract

Evaluating the change in gene expression is a common goal in many research areas, such as in toxicological studies, which are particularly important in pre‐clinical research. In practice, the analysis is often based on multiple *t*‐tests evaluated at the observed time points of the experiment, which limits the accuracy of determining the precise time at which the gene changes in expression. If a parametric approach is chosen, the analysis is often restricted to identifying the onset of an effect, but not its length. In this paper, we propose a parametric method to identify the time frame during which the gene expression significantly changes. This is achieved by fitting a parametric model and constructing a confidence band for its first derivative. The confidence band is derived by a two‐step bootstrap approach. It is summarized in terms of a hypothesis test, such that rejecting the null hypothesis means detecting a significant change in gene expression. Furthermore, a method for calculating confidence intervals for time points of interest (e.g., the beginning of significant change) is developed. We demonstrate the validity of our approach through a simulation study and present a variety of different applications to mouse gene expression data from a study investigating the effect of a Western diet on the progression of non‐alcoholic fatty liver disease.

## 1 Introduction

Understanding the mechanisms underlying disease responses is a key objective of toxicological studies, which are particularly important in preclinical research. A powerful tool in molecular biology for this purpose is RNA‐sequencing, based on the measurement of transcriptomes (Wang et al. 2009), which is integral to our understanding of genomic function (Stark et al. 2019). It measures the expressions of many genes simultaneously and is extensively used in drug discovery and development (Khatoon et al. 2014). One common objective in the field of RNA‐sequencing is to identify differentially expressed genes. Possible examples include dose‐gene expression data (Krug et al. 2013) or time‐gene expression data (Ghallab et al. 2021).

Anders and Huber (2010) introduced an approach to analyzing gene count data using the negative binomial distribution, that is employed in the well‐known DESeq R package. Its successor—the DESeq2 packages (Love et al. 2014)—has been cited over 35,000 times as of July 2024 (NCBI 1996). However, as described in the vignette of DESeq2 (Love et al. 2024), the analysis of the data includes the dose/time as a discrete covariate, which limits the precision of the analysis to the measured dose levels/time points.

One question, where this problem is particularly evident, is the identification of an alert concentration, defined as the lowest measured concentration where the difference between the concentration and the control significantly exceeds a critical level of the relevant response (Delignette‐Muller et al. 2011) or the lowest concentration with a noticeable effect (Jensen et al. 2019). Traditionally, the analysis is based on applying multiple t‐tests or—as recommended—Dunnett tests (Hothorn 2014), focusing on the measured dose levels. Consequently, with these tests it is not possible to identify a dosage between those levels. In order to address this issue, Kappenberg et al. (2021) proposed a model‐based approach assuming a monotonic relationship between dose and response. This method allows for a continuous identification of the lowest concentration at which the response significantly exceeds the response for the control by a given threshold. This method was further developed by Möllenhoff et al. (2022) through the introduction of a procedure that is independent of the assumption of monotonicity and replaces an asymptotic calculation of the variance by a parametric bootstrap.

When analyzing time‐response data, a similar problem is given by the identification of the time frame of significant changes in gene expression. For example, a change in gene expression indicates deregulation and thus the presence of a biological process. In a two‐group situation, a difference in time periods may indicate a slowing or acceleration of an underlying process. In addition, a gene ontology (GO) enrichment analysis of genes that undergo a change in expression over a given time period allows conclusions to be drawn about the order of processes occurring at the organism level, which can be used in particular to assess disease progression. In Ghallab et al. (2021) so called rest‐and‐jump genes (RJG) were observed. These are genes that showed delayed deregulation, either reaching a plateau afterwards or continuing to deregulate. In this context, it is of interest to consider the entire time frame during which the gene expression changes, rather than focusing only on the onset of an effect.

Based on this idea, in this paper we propose a model‐based approach that allows the identification of the entire time frame within which the change in gene expression is considered significant. This is accomplished by formulating a hypothesis test considering a test statistic based on the absolute value of the first derivative of a selected continuous parametric model or a B‐spline fit with regard to a threshold λ≥0. Rejecting the null hypothesis indicates the presence of a slope, that is significantly larger than λ and thereby a significant change in gene expression. Finally, the test decision is based on the construction of a lower simultaneous confidence band for the first derivative.

The proposed approach focuses on biologically significant changes in expression and the specific expression pattern of single or multiple genes. It therefore allows a more explicit interpretation of the results than, for example, classification by clustering (Androulakis et al. 2007). Especially in the case of toxicological data, recent publications suggest the use of parametric approaches (Kappenberg et al. 2023). The first derivative of a parametric model fit to the response data provides an understandable and easy to implement translation from the gene count scale to a measure of changes in expression. Similarly, the first derivative of a model fit utilizing B‐splines is itself a linear combination of a B‐spline basis (Fahrmeir et al. 2022). Both approaches are therefore well suited to analyzing expression change explicitly. The construction of simultaneous confidence bands presented here is specifically tailored to the first derivative, in contrast to established approaches that are fit to the response data (Neumann and Polzehl 1998; Wang and Yang 2010).

This paper is structured as follows: First, we introduce the method to estimate the time frame of significant change in gene expression, based on calculating the first derivative of the estimated time‐response model and the estimation of a corresponding lower simultaneous confidence band. Second, the methodology is subjected to a simulation study. Finally, potential applications are illustrated using a data set from the Western diet mice study (Ghallab et al. 2021), a toxicological serial‐sacrifice study (Dewanji 2005) that examines the impact of a Western diet (WD) on the progression of non‐alcoholic fatty liver disease (NAFLD).

## 2 Methodology

The fundamental idea of this method is to initially fit a model to the data and subsequently assess the change in gene expression by considering its first derivative. This is possible since the first derivative describes the slope of the gene expression curve at any time point t, and thus can be regarded as a measure for the change. Once a suitable model has been fitted, the next step is to develop a hypothesis test that tests if and when the first derivative significantly exceeds a chosen threshold, denoted λ. The corresponding time point marks a change in gene expression. The procedure is inspired by the method developed in Möllenhoff et al. (2022), adapted for the first derivative.

### 2.1 Model Fit and Derivation

We regard a data set with n observations in total, that are conducted at m different time points $t_{q}$, q=1,⋯,m. Let $n_{q}$ denote the number of observations at time point $t_{q}$, that is, $\sum_{q=1}^{m}n_{q}=n$ The presented method is designed to accommodate for repeated measurements. However, it can be simplified in case of an independent structure along t. In cases where there is a correlation between measurements at different time points, the same number of measurements, $n_{1}=\cdots =n_{q}$, are assumed for the purposes of simplicity. Furthermore, the study period is defined as $T=\left[t_{1},t_{m}\right]$ and the study duration as $\bar{t}=t_{m}-t_{1}$.

The initial step is to fit a parametric model to the data. We define

$$y_{p,q}=f\left(t_{q},\theta \right)+\varepsilon_{p,q}\;\;\;p=1,\cdots ,n_{q},\;\;\;q=1,\cdots ,m,$$

where f denotes the selected model. In order to incorporate possible correlations, we assume that the error terms $\varepsilon_{p}=\left(\varepsilon_{p,1},\cdots ,\varepsilon_{p,m}\right)$ are multivariate normally distributed $\varepsilon_{p}∼N_{m}\left(0,\Sigma \right)$ and independent for $p=1,\cdots ,n_{q}$. The parameters $\theta \in B\subset R^{r}$ are estimated by applying the maximum likelihood method. The likelihood is given by

$$L_{y,t}\left(\theta \right)=\prod_{p=1}^{n_{q}}\frac{\exp\left(-\frac{1}{2}{\left(y_{p}-f\left(t,\theta \right)\right)}^{T}\Sigma^{-1}\left(y_{p}-f\left(t,\theta \right)\right)\right)}{\sqrt{{\left(2\pi \right)}^{m}\det\left(\Sigma \right)}}$$

with $y_{p}=\left(y_{p,1},\cdots ,y_{p,m}\right)$ and $t=\left(t_{1},\cdots ,t_{m}\right)$. The estimator for the covariance matrix is given by

$${\hat{\Sigma }}^{2}=\frac{1}{n_{q}}\sum_{p=1}^{n_{q}}\left({\hat{\varepsilon }}_{p}-\hat{\bar{\varepsilon }}\right){\left({\hat{\varepsilon }}_{p}-\hat{\bar{\varepsilon }}\right)}^{T},$$

whereby the vector $\hat{\bar{\varepsilon }}=\left({\hat{\bar{\varepsilon }}}_{1},\cdots ,{\hat{\bar{\varepsilon }}}_{m}\right)$ consists of the means over all measurements

$${\hat{\varepsilon }}_{p,q}=y_{p,q}-f\left(t_{q},\hat{\theta }\right)\;\text{for}\;q=1,\cdots ,m.$$

In order to guarantee a good fit, the model is selected from a pool of possible models using a selection criterion, as, for example, the AIC (Fahrmeir et al. 2022).

As we are interested in detecting significant changes, the method developed here does not focus on the model f itself, but rather on its first derivative $f^{\prime }$. Motivated by the findings in Duda et al. (2022), we will focus on applying the method using the 4pLL (also called sigmoid Emax) model, the beta model, the exponential model and the quadratic model, respectively, as parameterized in Bornkamp et al. (2009). For details as well as the different first derivatives we refer to Supporting Information.

Another focus will be on utilizing B‐splines for model fitting as a more flexible alternative. As stated in Fahrmeir et al. (2022), a B‐spline basis function of degree l is constructed from (l+1) piecewise polynomials of degree l that are joined, such that the basis function is (l−1)‐times continuously differentiable. For the construction, the domain of the covariate, here time, is partitioned into intervals by so‐called knots. A full B‐spline basis consists of a set of the basis functions set up along these knots. The full model f is a linear combination of the basis. Naturally, since the basis functions are (l−1)‐times continuously differentiable, so is f. Therefore, B‐splines of degree l≥2 can be used to apply the proposed method. For details on the recursive definition of the B‐spline basis functions and their first derivatives, we refer to the Supporting Information.

Nevertheless, the method is applicable to all parametric models with a first derivative that can be calculated analytically.

### 2.2 Hypothesis Test for the Identification of Significant Changes

Here, we introduce a hypothesis test in which the rejection of $H_{0}$ leads to the assertion of a significant change in gene expression. In the previously introduced parametric framework this means that $f^{\prime }$ exceeds a threshold λ≥0 at some time point *t*, which leads to the hypotheses

(1) $$\begin{array}{c} H_{0}:\;\forall t\in T\;|f^{\prime }\left(t,\theta \right)|\leq \lambda \;\text{versus}\;H_{1}:\;\exists t\in T\;|f^{\prime }\left(t,\theta \right)|>\lambda . \end{array}$$

Rejecting $H_{0}$ implies the existence of at least one time point at which a change in gene expression is detected that exceeds the critical value λ. It is important to note that λ should be selected beforehand. Should any change in gene expression be of interest, λ can be set to 0. However, if only a larger change may be biologically relevant, λ should be adjusted accordingly in consultation with experts. For instance, regarding the absolute difference of the model fit to $\log_{2}$‐transformed counts at two time points $t_{a}$ and $t_{b}$, that is, $\left|f\left(t_{a},\theta \right)-f\left(t_{b},\theta \right)\right|$, the corresponding threshold is often set to $\log_{2}\left(1.5\right)$ (Ghallab et al. 2021; Kappenberg et al. 2021; Möllenhoff et al. 2022), investigating whether the foldchange between $t_{a}$ and $t_{b}$ exceeds 1.5. Based on this, a possible choice would be given by $\lambda =\log_{2}\left(1.5\right)\times {\bar{t}}^{-1}$ for the test regarding $f^{\prime }$, representing the slope of a straight line that increases by $\log_{1}\left(1.5\right)$ between $t_{1}$ to $t_{m}$. Of note, $\bar{t}$ is interchangeable with any other time frame, for example, $\left(1/2\right)\bar{t}$. Then the question is whether there is an absolute change in $\log_{2}\left(1.5\right)$ over half of the study period.

Next, let $L^{\alpha }\left(t,\hat{\theta }\right)$ denote a (1−α) lower simultaneous confidence band of $\left|f^{\prime }\left(t,\theta \right)\right|$, that is

$$P\{\forall t\in T:L^{\alpha }\left(t,\hat{\theta }\right)\leq |f^{\prime }\left(t,\theta \right)|\}\geq 1-\alpha .$$

According to Möllenhoff et al. (2022), rejecting $H_{0}$ in (1) if $L^{\alpha }\left(t,\hat{\theta }\right)>\lambda$ for any t∈T, leads to an α‐level test, as

$$\begin{array}{cc} & P_{H_{0}}\{\exists t\in T:L^{\alpha }\left(t,\hat{\theta }\right)>\lambda \}\\ & \;\leq P_{H_{0}}\{\exists t\in T:L^{\alpha }\left(t,\hat{\theta }\right)>|f^{\prime }\left(t,\theta \right)|\}\\ & \;=1-P_{H_{0}}\{\forall t\in T:L^{\alpha }\left(t,\hat{\theta }\right)\leq |f^{\prime }\left(t,\theta \right)|\}\\ & \;\leq 1-(1-\alpha )=\alpha . \end{array}$$

The time period of significant change in gene expression corresponds to the set of all t∈T for which $H_{0}$ in Equation (1) can be rejected, which we denote by

$$\tilde{S}≔\{t:L^{\alpha }\left(t,\hat{\theta }\right)>\lambda \}.$$

**FIGURE 1:**
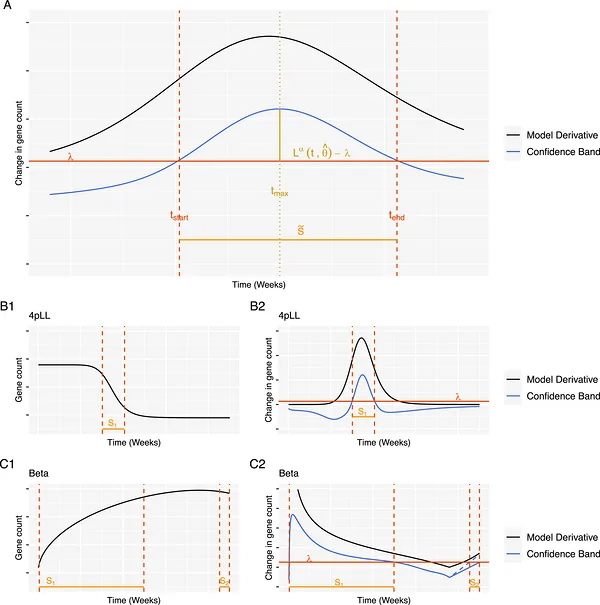
(A) Visualization of the time period of significant change in gene expression $\tilde{S}$ (orange) with two time points of interest. The red dotted lines indicate the beginning and end of $\tilde{S}$. The yellow line indicates the time point of maximum change in expression. $\tilde{S}$ is partitioned into coherent subsets if the gene expression curve follows a 4pLL model (B) or a beta model (C). B1 and C1 depict the fitted model, B2 and C2 the corresponding first derivative and confidence bands such that $H_{0}$ in Equation (1) is rejected.

Figure 1A depicts an illustrative example of a first derivative of a gene expression curve $f^{\prime }$ (black) and its lower confidence band (blue). The orange line represents the time period $\tilde{S}$, during which the confidence band exceeds the threshold λ, indicated by the red line.

While we can conclude significant changes in gene expression for all t∈T which fulfill $L^{\alpha }\left(t,\hat{\theta }\right)>\lambda$, some specific time points might be of higher interest. An example are the beginning and end of a significant change. Precisely, these are the first time point $t_{\text{start}}\in T$ with $L^{\alpha }\left(t_{\text{start}},\hat{\theta }\right)>\lambda$ and the last time point $t_{\text{end}}\in T$ with $L^{\alpha }\left(t_{\text{end}},\hat{\theta }\right)>\lambda$. Figure 1A indicates both $t_{\text{start}}$ and $t_{\text{end}}$ by the red vertical lines and thus also the period of significant change $\tilde{S}$. Another example is the time point of maximal change, indicated by the yellow vertical line in Figure 1A, representing the time point $t_{\max}\in T$ at which $L^{\alpha }\left(t,\hat{\theta }\right)-\lambda$, $t\in \tilde{S}$ assumes its (locally) maximal value.

These time points of interest are not necessarily unique. In case of a non‐monotonous function f (e.g., beta model), the confidence band can dip below the threshold λ and than exceed it again after a time period of no significant change. Therefore, we can partition S into ascending coherent subsets $\tilde{S}=\left(S_{1},\cdots ,S_{s}\right)$, meaning that for all j=1,⋯,s−1, there exists a $t^{\prime }\in T$ between $S_{j}$ and $S_{j+1}$ such that $L^{\alpha }\left(t^{\prime },\hat{\theta }\right)<\lambda$ and $\max\left(S_{j}\right)<\min\left(S_{j+1}\right)$. We refer to Figure 1B,C for a visual representation. Figure 1B depicts a gene expression curve that follows a 4pLL model on the left and its first derivative with a possible confidence band on the right. Here, $H_{0}$ is rejected and the time period of significant change in gene expression is coherent. Then $\tilde{S}$ equals $S_{1}$. Figure 1C depicts a beta model in the left and its first derivative with two possible confidence bands on the right. The solid line represents the scenario in which the decline in gene count in the latter part of the study period is not statistically significant. The dashed line on the other hand represents the scenario in which the aforementioned decline is significant. Here, $\tilde{S}$ splits into the coherent sub‐periods $S_{1}$ and $S_{2}$. Each $S_{j}$, j=1,⋯,s, has a unique set of the aforementioned time points of interest. In the following, we will focus on $t_{\text{start}}$ and $t_{\text{end}}$ as an example. Therefore, the set of times to search for is given by

(2) $$\begin{array}{c} \{t_{\text{start}}^{\left(j\right)}=\min\left(S_{j}\right):j=1,\cdots ,s\}\cup \{t_{\text{end}}^{\left(j\right)}=\max\left(S_{j}\right):j=1,\cdots ,s\}. \end{array}$$

The precise estimation of these quantities depends on the accuracy of the estimation of $L^{\alpha }\left(t,\hat{\theta }\right)$, which is described in the next section.

### 2.3 Estimating a Confidence Band of $\left|f^{\prime }\left(t\right)\right|$

In order to estimate a lower simultaneous confidence band $L^{\alpha }\left(t,\hat{\theta }\right)$ of $\left|f^{\prime }\left(t,\theta \right)\right|$ the method developed in Möllenhoff et al. (2022) is adjusted to the first derivative and to account for a dependence structure along the time axis. Therefore, we define

$$L^{\alpha }\left(t,\hat{\theta }\right):=|f^{\prime }\left(t,\hat{\theta }\right)|-c{\hat{\sigma }}_{|f^{\prime }\left(t,\hat{\theta }\right)|}$$

and estimate the critical value c, such that

$$P\{\max_{t\in T}\frac{\left|f^{\prime }\left(t,\hat{\theta }\right)|-|f^{\prime }\left(t,\theta \right)\right|}{{\hat{\sigma }}_{|f^{\prime }\left(t,\hat{\theta }\right)|}}\leq c\}=1-\alpha .$$

To estimate both ${\hat{\sigma }}_{|f^{\prime }\left(t,\hat{\theta }\right)|}$ and c, a two‐step parametric bootstrap procedure inspired by the classic bootstrap‐t method (Efron and Tibshirani 1994) has been proposed in Algorithm 1 by Möllenhoff et al. (2022) and is applied here by substituting the difference for the first derivative. In short, $\hat{\theta }$ and $\hat{\Sigma }$ are estimated from the original data set and used to generate $B_{1}$ bootstrap samples. This is done by randomly drawing iid. errors $\varepsilon_{p}^{*}=\left(\varepsilon_{p,1}^{*},\cdots ,\varepsilon_{p,m}^{*}\right)∼N_{m}\left(0,\hat{\Sigma }\right)$ and applying the formula

$$y_{p,q}^{*}=f\left(t_{q},\hat{\theta }\right)+\varepsilon_{p,q}^{*}\;\text{for}\;p=1,\cdots ,n_{q}\;\text{and}\;q=1,\cdots ,m.$$

These can be considered the first level of the bootstrap. For each of the generated samples, ${\hat{\theta }}_{l}^{*}$, $l=1,\cdots ,B_{1}$, are estimated and used to generate another $B_{2}$ bootstrap samples, which constitute the second level of the bootstrap. Then, ${\hat{\sigma }}_{|f^{\prime }\left(t,\hat{\theta }\right)|}$ is estimated from the first bootstrap level by calculating the empirical variance of the sample $\left|f^{\prime }\left(t,{\hat{\theta }}_{1}^{*}\right)|,\cdots ,|f^{\prime }\left(t,{\hat{\theta }}_{B_{1}}^{*}\right)\right|$. Similarly, ${\hat{\sigma }}_{|f^{\prime }\left(t,{\hat{\theta }}_{l}^{*}\right)|}$, $l=1,\cdots ,B_{1}$, is calculated for each of the generated first‐level bootstrap samples using the second‐level bootstrap samples. With these values

$$D^{*,l}:=\max_{t\in T}\frac{\left|f^{\prime }\left(t,{\hat{\theta }}_{l}^{*}\right)|-|f^{\prime }\left(t,\hat{\theta }\right)\right|}{{\hat{\sigma }}_{|f^{\prime }\left(t,{\hat{\theta }}_{l}^{*}\right)|}}$$

can be calculated for each $l=1,\cdots ,B_{1}$. The respective empirical (1−α)‐quantile of the distribution yields an estimate of the critical value c. A visual representation of this process is provided in the blue rectangle in Figure S10 (see Supporting Information).

## 3 Simulation Study

### 3.1 Setting and Data Generation

In order to evaluate the performance of our newly proposed method, a simulation study was conducted. Motivated by Möllenhoff et al. (2022) and Kappenberg et al. (2021) we focused on the 4pLL and the beta model as representatives of the models discussed in Section 4. This choice was also based on the findings regarding the 4pLL/sigmoid Emax model in Duda et al. (2022). The beta model was chosen as a suitable non‐monotonous alternative. The simulation settings were inspired by the Western diet mice trial discussed in Section 4. Precisely, we used the parameters obtained from the expression of the genes ‘Cd163’ (4pLL model, see Section 4.1 for details) and ‘Fam83a’ (beta model) in WD‐fed mice.

**FIGURE 2:**
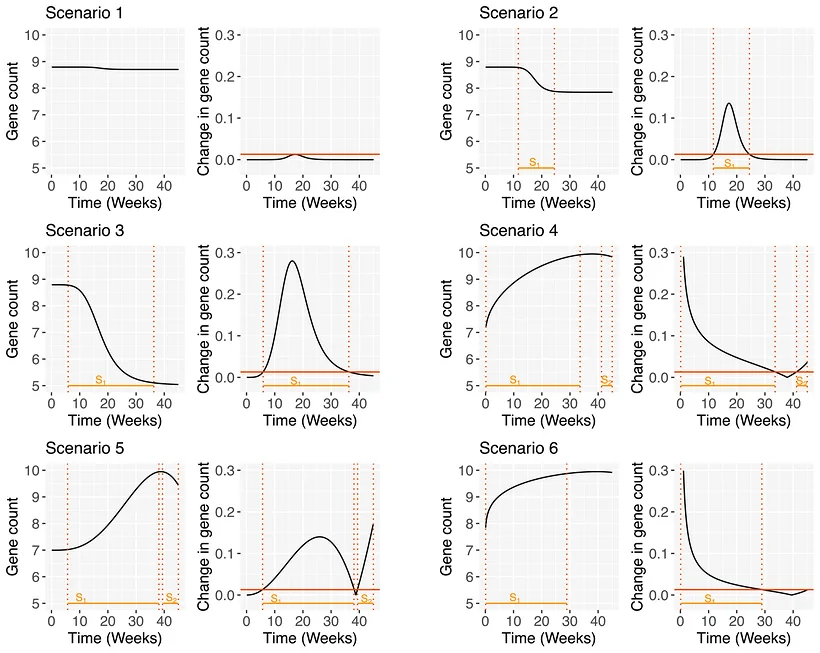
Visualization of the different scenarios. The horizontal red line indicates the relevance threshold $\lambda =\log_{2}\left(1.5\right)\times 45^{-1}$. Each case depicts the true model on the left and the true first derivative on the right. The vertical red lines mark the time frame of relevant change in regards to λ.

This resulted in the six basic scenarios, which are summarized in Table 1 and depicted in Figure 2.

**TABLE 1:** Overview of simulation scenarios.

Sce.	Short name	Model	Parameters θ	Description
1	No relevant change	4pLL	a=8.791, b=−0.089	Never exceeds λ=0.0130
			c=17.589, h=10.000	Reflects the margin of $H_{0}$.
2	Small jump	4pLL	a=8.791, b=−0.946	Exceeds λ=0.0130
			c=17.589, h=10.000	$\tilde{S}=\left(11.7,24.5\right)$
3	Large jump	4pLL	a=8.791, b=−3.783	Exceeds λ=0.0130
			c=17.589, h=5.000	$\tilde{S}=\left(5.9,36.3\right)$
4	Dip	beta	a=6.997, b=2.952	Exceeds λ=0.0130 twice
			$\delta_{1}=0.506,\delta_{2}=0.215$	$S_{1}=\left(0,33.6\right)$, $S_{2}=\left(41.2,45\right)$
5	Dip alternative	beta	a=6.997, b=2.952	Exceeds λ=0.0130 twice
			$\delta_{1}=3.286$, $\delta_{2}=1.290$	$S_{1}=\left(5.7,38.1\right)$, $S_{2}=\left(39.4,45\right)$
6	No dip	beta	a=6.997, b=2.952	Exceeds λ=0.0130
			$\delta_{1}=0.228$, $\delta_{2}=0.084$	$\tilde{S}=\left(0,28.9\right)$

Scenarios 1–3 were generated using the 4pLL model, while the latter three were generated with the beta model. In Scenario 1, there is no relevant change in gene expression respective to the threshold $\lambda =\log_{2}\left(1.5\right)\times 45^{-1}\approx 0.0130$. Therefore, $H_{0}$ in (1) cannot be rejected. In all other scenarios, there is a change in expression larger than λ, and, therefore, these scenarios correspond to the situation under the alternative. Scenario 4 reflects the unaltered parameters as estimated. Scenarios 2, 3, 5 and 6 were based on the real examples, but changed to reflect a different slope, see Table 1.

The observations were simulated at nine time points (0, 3, 9, 15, 21, 27, 33, 39 and 45 weeks) in accordance with the feeding times observed in the Western diet mice trial, following the beginning of the study. A total of 47 data points were simulated consisting of 5 mice at the first 7 time points and 4 and 8 mice at 39 and 45 weeks, respectively.

Of note, the data set used for the simulation was derived from a toxicological study that required the sacrifice of the mice in order to obtain measurements. In order to evaluate the disease progression, sacrifices were made at different time points. Consequently, each measurement represents a distinct mouse and we can assume independence across the measurements. Such studies are referred to as serial‐sacrifice experiments (Dewanji 2005; Bergman and Turnbull 1983) and will be the focal point of the ensuing sections. As previously stated in Section 2.1, the procedure can then be simplified by assuming the error terms to be independent normally distributed $\varepsilon_{p,q}∼N\left(0,\sigma^{2}\right)$. Given the relatively small number of observations (n=47) we will apply the unbiased estimator for the variance given by

$${\hat{\sigma }}^{2}=\frac{1}{n-r}\sum_{p=1}^{m}\sum_{q=1}^{n_{p}}{\left\{y_{p,q}-f\left(t_{p},\hat{\theta }\right)\right\}}^{2}.$$

In order to gain a comprehensive understanding of the impact of the standard deviation, five distinct levels for each of the six basic scenarios were considered: ‘small’, ‘mid‐small’, ‘medium’, ‘mid‐large’ and ‘large’. The ‘medium’ standard deviation of the realistic Scenario 4 is as estimated from the real world data. For all other scenarios, the ‘medium’ standard deviation was calculated via linear transformation with regard to the respective maximum absolute differences in gene expression compared to the realistic example of the same model type. Regarding the parameterization introduced in Section 2.1, the maximum absolute value corresponds to the parameter b∈θ. Accordingly, we designate $b_{\text{sce}}\in \theta_{\text{sce}}$ as the maximum absolute difference of the scenario and $b_{\text{real}}\in \theta_{\text{real}}$ as the maximum absolute difference estimated from the example. With this, we obtained the transformed standard deviation by $\sigma_{\text{sce,medium}}=b_{\text{sce}}\times b_{\text{real}}^{-1}\times \sigma_{\text{real}}$, where $\sigma_{\text{real}}$ the estimated value from the real world data.

From these ‘medium’ values of standard deviation, the ‘small’, ‘mid‐small’, ‘mid‐large’ and ‘large’ levels were calculated by multiplying with 0.5, 0.75, 1.5 and 2, respectively, see Table S1 in the Supporting Information for details.

Given either the model function, f, of the beta model or the 4pLL model and the values of θ and σ, the gene expression data was simulated by drawing iid. errors $\varepsilon_{p,q}^{\text{sim}}∼N\left(0,1\right)$ and applying the formula $y_{p,q}^{\text{sim}}=f\left(t_{p},\hat{\theta }\right)+\hat{\sigma }\varepsilon_{p,q}^{\text{sim}}$ for p=1,⋯,m and $q=1,\cdots ,n_{p}$. As described in Möllenhoff et al. (2022) and following the postulations from Efron and Tibshirani (1994) we chose a large value for the estimation of the quantiles by the first‐level bootstrap and a relatively small value for the estimation of the standard error by the second‐level bootstrap. Precisely, we chose $B_{1}=500$ and $B_{2}=25$ repetitions. Smaller values would lead to imprecise estimations, whereas larger values could improve the results, but would require excessive computational effort. Each procedure was repeated in 1000 simulation runs. Outcomes of the simulation study were the number of rejections of $H_{0}$ in (1) and the identified time frame of significant change in gene expression. The majority of the code was run on R version 4.3.2 using a Windows 11 ×64 PC. The simulation scenarios were run on a HPC‐cluster employing R version 4.3.1. The runtime of each simulated study on a commercially available Windows PC is approximately 5 min, with a variability of a few minutes depending on the number of CPU cores.

### 3.2 Results

#### 3.2.1 Rejection of $H_{0}$

**FIGURE 3:**
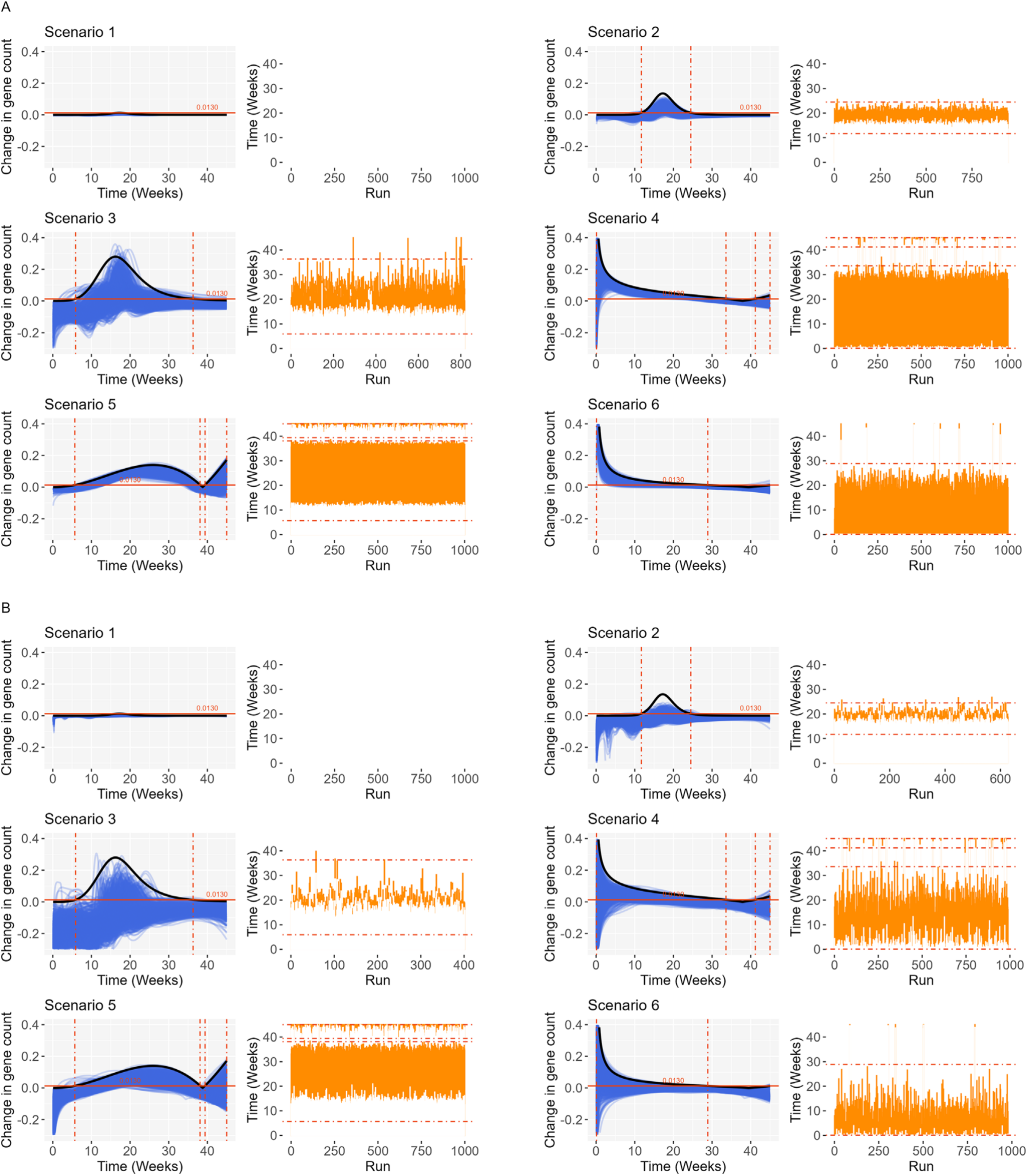
Results of the six simulation scenarios for a mid‐small (A) and mid‐large (B) standard deviation. The left column depicts the true first derivative and the estimated confidence bands. The right column depicts the estimated time frames of significant change in gene expression for each run.

Table 2 shows the number of rejections of $H_{0}$ in 1000 runs for all configurations. The left column in Figure 3 depicts the estimated confidence bands for a mid‐small and mid‐large standard deviation. We refer to the Supporting Information Figures S13–S15 for the other cases.

**TABLE 2:** Number of rejections of $H_{0}$ in 1000 runs.

σ level	Sce. 1	Sce. 2	Sce. 3	Sce. 4	Sce. 5	Sce. 6
small	0	999	1000	1000	1000	1000
m.‐small	0	946	818	1000	1000	1000
medium	0	830	514	1000	1000	1000
m.‐large	0	627	401	989	999	978
large	0	372	303	814	957	780

In Scenario 1, no false rejection of $H_{0}$ was observed in any of the cases, indicating a type I error of 0. This scenario corresponds to the margin of the null hypothesis in Equation (1), here given by λ=0.0130. The conservatism of the test decision even for a large standard deviation may therefore be partially attributed to the effect being very small and an overall tendency to slightly underestimate the time frame of significant change in expression discussed in the next subsection.

In general, our proposed method shows high power when the beta model is the true model. Up to a medium standard deviation, $H_{0}$ is always rejected for Scenarios 4–6. Even for a large standard deviation, the power remains at about 80% for Scenarios 4 and 6 and above 95% for Scenario 5. Accordingly, the estimated confidence bands show a low variability.

For the remaining scenarios generated with the 4pLL model—Scenarios 2 and 3—we observe a large influence of the standard deviation. In particular, the power is above 80% for a small and mid‐small variability, but lies between 30% and 65% for a large and mid‐large variability, respectively (see Table 2). This influence seems to be more pronounced for the larger jump in gene expression simulated in Scenario 3. In this case, only 514 of significant changes were detected for a medium standard deviation.

It is noteworthy that the (mid‐)large standard deviations (see Table S1 in the Supporting Information) employed in the generation of Scenarios 2 and 3 is considerably large compared to the absolute change in gene expression. Naturally, this leads to a high variability in the generated simulation studies (see Figure S11 in the Supporting Information). However, we observe that in the case of the 4pLL model, the translation of a high degree of variability on the count scale leads to an extreme degree of variability on the scale of the change in gene expression considered when applying the first derivative (see Figure S12 in the Supporting Information). It is important to interpret the results presented in light of these considerations. It is also important to note that the same effect occurs when generating the first and second level bootstrap runs. This is likely to have an impact on the estimation process, especially if the data set has a high degree of variability.

For Scenarios 2 and 3 we note a visible crater in the estimated confidence bands around the week where the true first derivative reaches its maximum, which corresponds to the inflection point of the model function (see Figure 3 and Figures S13–S15 in the Supporting Information). While the extent is likely to be at least partially attributable to the size of the standard deviation, the trend is discernible across all cases. Given that the effect appears to be more pronounced in Scenario 3 than in Scenario 2, it is plausible that its magnitude may correlate with the steepness of the true model function in the area around its inflection point. This conclusion is supported by the absence of a comparable crater for Scenario 5, the only scenario with an inflection point generated using the beta model, which is markedly less steep than Scenarios 2 and 3.

#### 3.2.2 Estimated Time Frame of Significant Change in Gene Expression

While the number of rejections of $H_{0}$ is largely satisfactory, a reliable detection of the time frame of significant change in gene expression is of greater interest. Therefore, we considered the bias and variance of each start/end time point of the estimated time frame of significant change in gene expression. Furthermore, we examined the number of runs in which the true number of coherent subsets of $\tilde{S}$ was correctly identified. We refer to Table S2 in the Supporting Information for a summary of all results. As anticipated, the true number of coherent subsets was consistently detected for Scenarios 2 and 3 if $H_{0}$ was rejected. Given the monotonicity of the 4pLL model, the time frame of significant change in expression is, by definition, continuous. However, as described above, a crater was observed in some estimated lower simultaneous confidence bands. In a small number of cases, this resulted in the identification of two disjointed subsets. Overall, the number of these cases is less than 12 for Scenario 2. In case of Scenario 3, the same is observed except for a mid‐small and medium standard deviation, which respectively fall within the ranges of 200 and 70 cases.

While $H_{0}$ was reliably rejected for Scenarios 4 and 5, the second time frame of significant change in expression was not always detected. The smaller dip in Scenario 4 was only identified approximately 120 times when the standard deviation was small and approximately 40 times otherwise. The more pronounced dip in Scenario 5 was detected 999 times for a small standard deviation, 671 times for a medium standard deviation and 260 times for a large standard deviation. However, it should be noted that in both scenarios, the maximum value in gene count is reached around week 39, which is the second to last sample time point. Therefore, the detection of the dip is contingent only on the last measurement. In Scenario 6, a dip at the end was incorrectly identified less than 15 times in all cases.

The right columns in Figures 3 depict the estimated time frames of significant change in gene expression for each of the 1000 simulation runs for the mid‐small and mid‐large standard deviations, respectively. We refer to Figures S13–S15 in the Supporting Information for the other cases. The true values are indicated by the red lines. In general, we note that the estimated start of the time frame of significant change in expression displays a positive bias for all scenarios, whereas the estimated end displays a negative bias. Therefore, the time frame is overall slightly underestimated. The bias depends largely on the standard deviation and the slope of the first derivative. Scenario 6 exhibits the smallest bias for the onset of the significant change at the beginning of the study, never exceeding 0.375 weeks. Scenario 4 is similarly flat. Its bias never exceeds 2.379. Scenario 5 is characterized by a steeper slope and exhibits a noticeably larger bias. The range is from 3.956 weeks for a small standard deviation to 8.865 weeks for a large standard deviation. For Scenarios 2 and 3—disregarding the crate when detected—we observe similar results. The influence of the slope seems to have a reverse effect on the bias of the end of the identified time frame of significant change. In particular, flat scenarios, such as Scenario 6, demonstrate a noticeably larger bias for all levels of standard deviation. In addition, the bias is overall larger than for the start of the significant change, with a range of −1.225 to −23.591 weeks.

In Scenarios 4 and 5, a second time frame of significant change in expression was simulated. Since in both cases it is cut off at the end of the study, we consider only the starts of the second subsets of $\tilde{S}$, respectively. Again, we note a positive bias, which ranges from almost 2 weeks to nearly 3.5 weeks. Finally, the variance shows a similar behavior to the bias.

## 4 Case Study

In this section, three possible applications of our method are presented. For illustration, we use data from the Western diet mice study (Ghallab et al. 2021). A total of 79 male mice were fed a Western (WD) or standard (SD) diet for a maximum of 48 weeks to assess the effect of a high fat diet on the development of non‐alcoholic fatty liver disease. Up to 8 mice per diet were sacrificed at 3,6,30,36,42 and 48 weeks, and WD‐fed mice were additionally sacrificed at 12,18 and 24 weeks. RNA was extracted from frozen liver tissue and RNA‐seq analysis was performed. Here, we use the pre‐processed data set, that was generated using Salmon (Patro et al. 2017) and the R packages tximeta (Love et al. 2020) and DESeq2 (Love, et al. 2014). The count data was normalized and $\log_{2}$‐transformed using the *vst()* function (Anders and Huber 2010; Huber et al. 2003; Tibshirani 1988) from the DESeq2 package. As the initial samples were collected at 3 weeks, the beginning of the study at t=0 in the subsequent analysis represents these initial data points.

The first example illustrates the application of the method to four genes expressing different shapes. For each gene, the methodology is demonstrated using a parametric model fit as well as fitting B‐splines. In the second example, we select a pool of nearly 10,000 genes and use our method to identify those that change significantly between 10 and 25 weeks. A GO enrichment analysis is then performed on the identified genes. In all cases, the two‐step bootstrap procedure was implemented with $B_{1}=500$ and $B_{2}=25$ bootstrap runs as described in Section 3.1.

### 4.1 Application to an Individual Gene

**FIGURE 4:**
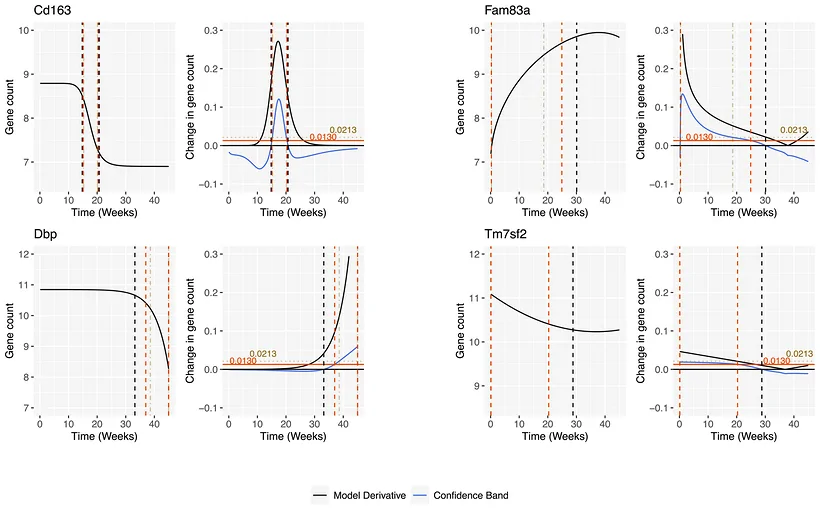
On the left is a depiction of the fit of the best parametric model to the normalized gene count over time for each of the four example genes. On the right, the first derivative and the estimated confidence band of the model fit are displayed. The horizontal red line indicates $\lambda =\log_{2}\left(1.5\right)\times 45^{-1}$. The vertical dashed red lines indicate the corresponding time frame of significant change in gene expression. The yellow dotted lines correspond to an alternative choice of $\lambda =\log_{2}\left(1.5\right)\times 27.5^{-1}$. The black lines represent the case λ=0. The expression of the gene ‘Cd163’ follows a 4pLL model (upper left), the expression of the gene ‘Fam83a’ follows a beta model (upper right), the expression of the gene ‘Dbp’ follows an exponential model (lower left) and the expression of the gene ‘Tm7sf2’ follows a quadratic model (lower right).

For this example we applied the proposed method to four individual genes measured in WD‐fed mice. The method was implemented using two different types of fit: a parametric model fit and a B‐spline fit to the data. Firstly, the results obtained from the application of the parametric models will be presented. In a preceding model selection step, the 4pLL model, the beta model, the exponential model and the quadratic model were fitted to the gene expression data, respectively. The chosen examples are such that each of the parametric models is represented by one of the genes. Precisely, the 4pLL model is illustrated by the gene ‘Cd163’ (upper left), the beta model is illustrated by the gene ‘Fam83a’ (upper right), the exponential model is illustrated by the gene ‘Dbp’ (lower left) and the quadratic model is illustrated by the gene ‘Tm7sf2’. The left panels of each gene in Figure 4 depict the respective estimated gene expression curves.

In Ghallab et al. (2021) both ‘Cd163’ and ‘Fam83a’ were identified as RJG. This implies that the gene in question is only deregulated after a specific period of time, in these cases, after 24 and 18 weeks, respectively. According to identification as an RJG, it appears that approximately 10–12 weeks pass before the gene ‘Cd163’ is down‐regulated. Following an additional 10–12 weeks, the deregulated expression seems to reach a constant value. In contrast, the gene ‘Fam83a’ appears to become deregulated immediately. After week 20–30, the deregulated expression seems to reach a constant value. Although not identified as RJG in Ghallab et al. (2021), the ‘Dbp’ gene appears to become down‐regulated only after a period of 30–35 weeks. In contrast, the ‘Tm7sf2’ gene appears to be deregulated at the beginning of the study, reaching a plateau after week 30.

In the respective right panels of Figure 4, the corresponding first derivatives and the estimated confidence bands are depicted. The various horizontal lines represent different potential choices for the threshold λ, while the corresponding vertical lines represent the respective time frames of significant change in gene expression. The black line represents the case where λ=0, while the red line corresponds to the aforementioned case $\lambda =\log_{2}\left(1.5\right)\times 45^{-1}=0.0130$ (see Section 2.2). Similarly, a third threshold was calculated, indicated by the yellow line, which corresponds to a change of $\log_{2}\left(1.5\right)$ in gene expression over the span of half the study. This third threshold is given by $\lambda =\log_{2}\left(1.5\right)\times 27.5^{-1}=0.0213$.

We observe that $H_{0}$ is rejected for all genes and all thresholds, with the exception of the gene ‘Tm7sf2’. For this gene, a significant change cannot be concluded at the threshold of λ=0.0213. In the following, we will present a summary of the analysis for $\lambda =\log_{2}\left(1.5\right)\times 45^{-1}$ as an illustrative example. For a detailed analysis of all four genes we refer to the Supporting Information. Confidence intervals for the time points of interest were estimated using a third‐level bootstrap (for details on the exact procedure we refer to the Supporting Information).

The period during which the estimated significant changes in gene expression of ‘Cd163’ occurs begins at 15.1 weeks (95% CI $\left[13.2,17.8\right]$) and ends at 20.3 weeks (95% CI $\left[17.4,23.5\right]$). The ‘Dbp’ gene shows similar delayed deregulation. The estimated time frame of significant changes in gene expression begins at 35.4 weeks (95% CI $\left[29.3,37.0\right]$) and continues until the end of the study at 45 weeks (95% CI $\left[41.4,45.0\right]$). Consequently, we can conclude the classical RJG form for both genes. For the gene ‘Fam83a’, the estimated time period begins at 0.3 weeks (95% CI $\left[0.1,1.9\right]$) and ends at 24.9 weeks (95% CI $\left[15.7,32.1\right]$). Similarly, the time frame of significant change in expression estimated for the gene ‘Tm7sf2’ begins at the start of the study (95% CI $\left[0.1,14.0\right]$) and ends at 20.3 weeks (95% CI $\left[12.2,25.6\right]$). Therefore, we would not conclude an RJG form in both cases.

Additionally, we applied the proposed method using B‐splines to all four genes and examined the influence of both the degree of the basis functions and the number of knots. Precisely, we conducted the analysis using the R packages splines2 (Wang and Yan 2021, 2025), which includes the derivatives of the basis functions. The degree of the basis functions was selected to be l∈{2,3} with either two or three internal knots, which were automatically set at suitable quantiles (Wang and Yan 2025). Basis functions of higher degree and a greater number of knots were considered, but overall resulted in overfitting for the given data. We refer to Figure S1 in the Supporting Information for a depiction of the basis functions and their first derivatives. After calculating the values of the B‐spline basis using the R function *bSpline()*, a linear model was fitted using the *lm()* function. The first derivatives were calculated via the function *dbs()*.

For the gene ‘Cd163’, the results obtained are mostly comparable to those produced by fitting parametric models. The time frame of significant change overlaps for all cases. In case of l=2 degrees with two knots, the results for the gene ‘Fam83a’ are also very similar to the parametric case. However, for all other B‐spline cases, we detect a period of inactivity around 10 weeks. This phenomenon can be attributed to the flexible nature of B‐splines and the noticeable dip in expression presented by the measures at week 9. However, the estimated time frames for significant changes in gene expression are found to overlap satisfactorily for these three cases, reinforcing the robustness of the results obtained. For the gene ‘Dbp’ two distinct time frames of significant change in expression were detected. While the latter period is comparable to the estimated period applying the parametric approach, the earlier period is not detected, possibly indicating an insufficient fit of the exponential model and thereby the collection of parametric models. Interestingly, we detect no significant change for the expression of the gene ‘Tm7sf2’. However, the detected signal found utilizing a parametric fit is very small (see Figure S7 in the Supporting Information). This suggests that the parametric approach may be slightly more sensitive when the parametric model fits the data well.

The findings indicate that with increasing degree and number of knots, the estimated confidence bands become more wavy in nature, as is also observed for the model fit applying B‐splines. This noticeable structure is clearly influenced by the placement of the B‐spline basis functions along the knots and is expected due to the definition. We note that for the three genes ‘Cd163’, ‘Fam83a’ and ‘Dbp’ the estimated time frames are the widest for l=2 degrees with two knots. However, it is not possible to make a similar assertion regarding the remaining cases. In general, the correspondence of the fits indicates that core regions of activity can be detected by the proposed method, irrespective of the specified preliminaries.

**FIGURE 5:**
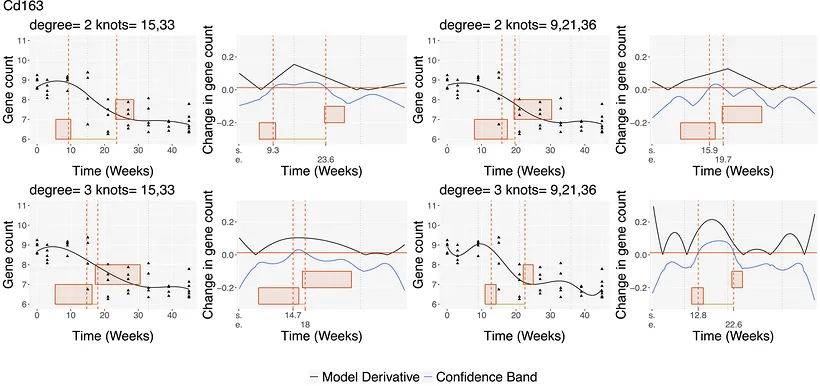
The fit of B‐splines to the normalized gene count of the gene ‘Cd163’ over time is depicted on the left for each presented combination of degree and number of knots. The degree of the basis functions and the knot placement are stated in the subtitle of the respective plots. Additionally, the knots are indicated by gray dotted lines. On the right, the first derivatives and the estimated confidence bands are displayed. The horizontal red lines indicate $\lambda =\log_{2}\left(1.5\right)\times 45^{-1}$. The vertical dashed red lines and the horizontal yellow lines indicate the corresponding time frames of significant change in gene expression. The red boxes illustrate the estimated confidence intervals for the respective time points of interest.

Figure 5 depicts the different fits of B‐splines for the gene ‘Cd163’ on the left and their respective first derivatives and estimated confidence bands on the right. As before, the red horizontal lines indicate the threshold λ=0.0130. The red vertical lines indicate the estimated periods of change in gene expression. In general, the fits with B‐splines appear less steep than the parametric fit with the 4pLL model. In accordance with this observation, a significant change in gene expression was detected from 9.3−16.2 weeks until 17.2−22.4 weeks. This time frame is slightly broader for degree l=2 with two knots or l=3 with three knots than the outcomes derived from employing the parametric approach.

We refer to the Supporting Information for an analysis of the results obtained by fitting B‐splines to the three other genes. Moreover, an application for a gene that deregulates in both diet groups and a subsequent comparison of the estimated time frames of significant changes in gene expression is included in the Supporting Information.

### 4.2 GO Enrichment Analysis

For this example we applied our proposed method to identify a subset of differentially expressed genes that deregulate during a pre‐selected time period (10 to 25 weeks). In a subsequent step, we conducted a GO enrichment analysis on these genes. This means, we examined if a significant number of the identified genes were assigned to pre‐specified GO groups (Consortium 2001), in which the genes were grouped according to their connection to biological functions. This approach would permit a practitioner to draw inferences regarding the time frame within which specific biological processes occur. For instance, this could be used to evaluate the progression of a disease.

To achieve this, we selected 9881 genes that were identified as differentially expressed in Ghallab et al. (2021) comparing SD‐fed mice at week 0 (time on study) and WD‐fed mice at week 45.

**FIGURE 6:**
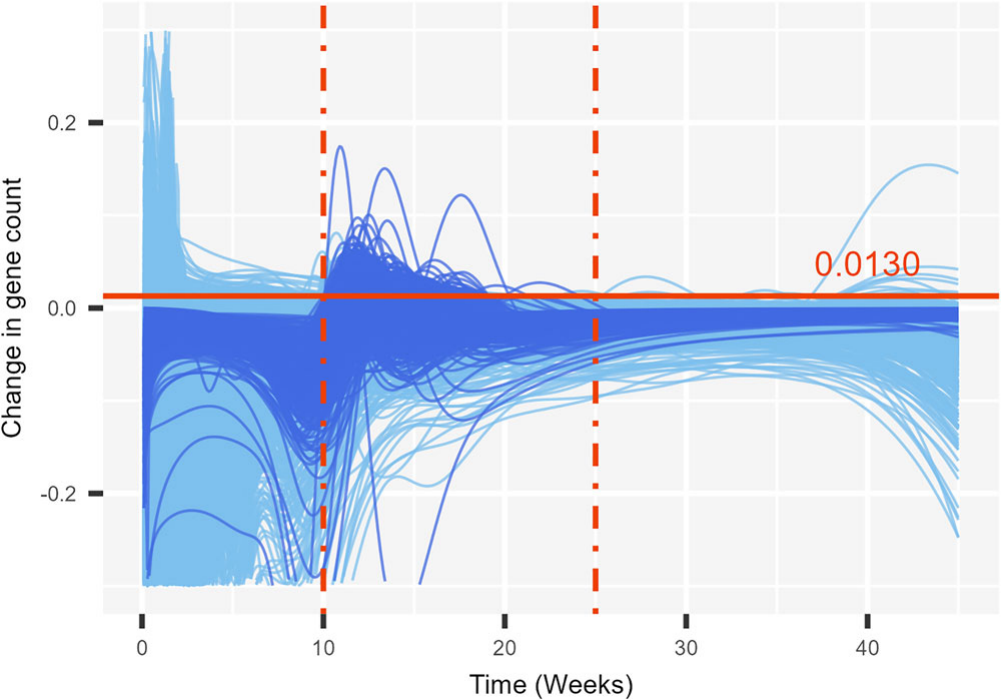
Estimated confidence bands of the 9881 pre‐selected genes. The darker blue lines correspond to the genes for which the estimated period of significant change in expression was determined to be between 10 and 25 weeks.

We assumed that the 4pLL model was the most appropriate for the analysis and applied our method to identify the time periods of significant change in gene expression. Figure 6 depicts the estimated confidence bands. The results were then employed to identify the genes with an estimated time period of significant change in expression between weeks 10 and 25. A total of 569 genes were identified.

A GO enrichment analysis was conducted on the aforementioned genes using the topGO R package (Alexa and Rahnenführer 2024; Alexa et al. 2006). In essence, Fisher's exact test is employed to verify whether a number of interesting genes is annotated to a specific functional group that exceeds what would be expected by chance. The 20 gene ontology groups with the smallest adjusted *p*‐value are presented in Table S3 in the Supporting Information. For instance, the a significant number of genes was assigned to the GO group ‘inflammatory response’. This is in accordance with the findings that inflammation is a pivotal event in the progression of NAFLD (Ghallab et al. 2021; Sutti and Albano 2020). In Ghallab et al. (2021) few inflammatory foci were observed in week 3 and the number strongly increased after week 36. Lipogranulomas, single or multiple fat globules surrounded by chronic inflammatory cells and Kupffer cells (Brunt 2002), were observed as early as week 6 and increased at week 18.

## 5 Discussion and Conclusion

In this paper, we proposed a parametric method to estimate the time frame during which the expression of a gene changes significantly. Thereby, we have provided a tool that allows for an interpretation that is not limited to the onset of an effect or the observed time points and produces results based on a hypothesis test to the α‐level. As demonstrated by the 4pLL model, the beta model, the exponential model, the quadratic model and B‐splines, the approach is applicable to a wide range of potential models, with the singular restriction that the first derivative can be derived analytically from the model function. It can be employed to analyse the expression of a single gene or, in conjunction with a GO enrichment analysis, to infer biological function. Moreover, the methodology can be applied to other forms of time‐response or dose‐response data.

Of course, there are limits to the method. The simulation results discussed in Section 3.2 show a clear tendency to underestimate the time frame of significant change in expression. The effect is relatively modest for medium levels of data variability. However, it becomes more pronounced with greater variability. Overall, the method would benefit from a built‐in stabilization procedure against the predictable effects of a large standard deviation. Similarly, the noticeable effect of the form of the B‐spline basis functions on the estimated confidence band may complicate the interpretation of the results in cases of overfitting. One way to combat this is to include penalization in the method, such as by fitting P‐splines instead (see Fahrmeir et al. 2022). In addition, it would be interesting to explore the possibility of adapting alternative construction methods for simultaneous confidence bands to the first derivative. This is especially true for B‐splines, since the first derivative of the entire polynomial spline is again a linear combination of B‐spline basis functions. Therefore, established methods for the estimation of simultaneous confidence bands could be applied as an alternative, for example, Krivobokova et al. (2010) for penalized splines. Also, while we attempted to discern any relationship between the estimated time frames and knot placement in the case study, a thorough consideration of this topic would exceed the scope of this paper. However, it would be an interesting and important endeavor to expand this analysis in the future.

Often, a statistical conclusion on more than one gene expression curve is of interest. In Section 4.2, we presented a possible application to a large number of genes in combination with a GO enrichment analysis. Nevertheless, a multitude of potential biological questions may necessitate alternative approaches. This frequently gives rise to a multiple testing problem. Hence, our proposed method would highly benefit from the development of a method that corrects for this issue beyond a simple correction of the p‐values.

In conclusion, we posit that our proposed method is a valuable addition to the existing tools for the analysis of gene expression data. It allows for a comprehensive analysis over the entire study period and is applicable to a wide range of expression curves.

## Conflicts of Interest

The authors declare no conflicts of interest.

## Open Research Badges

This article has earned an Open Data badge for making publicly available the digitally‐shareable data necessary to reproduce the reported results. The data is available in the Supporting Information section.

This article has earned an open data badge “**Reproducible Research**” for making publicly available the code necessary to reproduce the reported results. The results reported in this article could fully be reproduced.

## Supporting information


**Supporting File 1:** bimj70181‐sup‐0001‐SuppMat.pdf.

**Supporting File 2:** bimj70181‐sup‐0002‐Data.zip.

## Data Availability

The case study data set is publicly available at the SRA database with reference number PRJNA953810.
