# Supplementary material for: Identification of Changes in Gene Expression
Source: Biom J. 2026 Sep 24;68(5):e70181. doi: 10.1002/bimj.70181 (PMC13602452; doi:10.1002/bimj.70181)
Supplement: Supplementary file 1 [file BIMJ-68-e70181-s002.pdf]

Supplementary material to: Identification of changes in gene expression

L. Ameis[1] and K. Möllenhoff[1*]

[1]Institute of Medical Statistics and Computational Biology (IMSB), Faculty of Medicine, University of Cologne

1 Parametric model functions and first derivatives

In the following section, we present the four parametric models used as examples in this paper, along with their respective first derivatives. The parameterizations and descriptions of the parameters are in accordance with the conventions noted in Bornkamp, B. and Pinheiro, J. and Bretz, F. (2009, 2017).

4pLL/sigEmax

E_0	Placebo effect
E_{max}	Asymptotic maximum change from placebo effect
ED_{50}	Dose giving half of the asymptotic maximum effect
h	Hill parameter, determining the steepness of the model at the ED50

$$f_{4pLL}(t, (E_0, E_{max}, ED_{50}, h)) = E_0 + E_{max}\frac{t^h}{ED_{50}^h + t^h}$$

$$f'_{4pLL}\left(t, (E_0, E_{max}, ED_{50}, h)\right) = \frac{E_{max} \times ED_{50}^h \times t^{h-1} \times h}{\left(ED_{50}^h + t^h\right)^2}$$

beta model

E_0	Placebo effect
E_{max}	Maximum effect
δ_1	Delta1 parameter
δ_2	Delta2 parameter

$$f_{beta}\left(t,(E_0,E_{max},\delta_1,\delta_2)\right) = E_0 + E_{max}B(\delta_1,\delta_2)\left(\frac{t}{scal}\right)^{\delta_1}\left(1-\frac{t}{scal}\right)^{\delta_2}$$

$$f'_{beta}\left(t,(E_0,E_{max},\delta_1,\delta_2)\right) = \frac{E_{max}\times B_\delta \times t^{\delta_1-1}\times\left(1-\frac{t}{scal}\right)^{\delta_2-1}\times(\delta_1\times scal-(\delta_1+\delta_2)\times t)}{scal^{\delta_1+1}}$$

Hereby, let $B_\delta = \left\{\,(\delta_1+\delta_2)^{\delta_1+\delta_2}\,\right\}\times\left\{\delta_1^{\delta_1}\times\delta_2^{\delta_2}\right\}^{-1}$ and $scal$ denote a fixed scaling parameter.

Exponential model

E_0	Placebo effect
E_1	E1 parameter
δ	Delta parameter, controlling the convexity of the model

$$f_{exp}(t,(E_0,E_1,\delta)) = E_0 + E_1\left(\exp\left(\frac{t}{\delta}\right)-1\right)$$

$$f'_{exp}(t,(E_0,E_1,\delta)) = E_1\times\left(\exp\left(\frac{t}{\delta}\right)\times\left(\frac{1}{\delta}\right)\right)$$

Quadratic model

E_0	Placebo effect
β_1	Beta1 parameter
β_2	Beta2 parameter, controls, whether model is convex or concave

$$f_{quad}(t,(E_0,\beta_1,\beta_2)) = E_0 + \beta_1 t + \beta_2 t^2$$

$$f'_{quad}(t,(E_0,\beta_1,\beta_2)) = \beta_1 + 2\beta_2 t$$

2 B-spline basis functions and first derivatives

Let the interval $[t_1, t_m]$ denote the study period, and let $t_1 = \kappa_1 < ... < \kappa_{\tilde{m}} = t_m$ be a suitable partition, e.g. equidistant or quantile based. We call κ_j, $j = 1, \dots, \tilde{m}$, the inner knots. As presented in Fahrmeir, L. and Kneib, T. and Lang, S. and Marx, B.D. (2022), the B-spline basis functions of degree l are recursively defined as follows. Let $d = l + \tilde{m} - 1$:

$$l = 0 \qquad B_j^0(t) = I(\kappa_j \leq t < \kappa_{j+1}) = \begin{cases} 1 & \kappa_j \leq t < \kappa_{j+1} \\ 0 & \text{otherwise,} \end{cases}$$

$$l > 1 \qquad B_j^l(t) = \frac{t - \kappa_{j-l}}{\kappa_j - \kappa_{j-l}} B_{j-1}^{l-1}(t) + \frac{\kappa_{j+1} - t}{\kappa_{j+1} - \kappa_{j+1-l}} B_j^{l-1}(t) \text{ for } j = 1, \dots, d.$$

Hereby, $I()$ denotes the indicator function. To ensure that this definition is well-defined for the entire study period, it is necessary to add $2l$ outer knots that extend outside the interval $[t_1, t_m]$. Fahrmeir, L. and Kneib, T. and Lang, S. and Marx, B.D. (2022) also provides the first derivative of a B-spline basis function of degree l:

$$\frac{\partial}{\partial t} B_j^l(t) = l \left(\frac{1}{\kappa_j - \kappa_{j-l}} B_{j-1}^{l-1}(t) - \frac{1}{\kappa_{j+1} - \kappa_{j+1-l}} B_j^{l-1}(t) \right)$$

Therefore, the following holds for f:

$$f(t) = \sum_j \gamma_j B_j^l(t) \implies f'(t) = l \sum_j \frac{\gamma_j - \gamma_{j-1}}{\kappa_j - \kappa_{j-l}} B_{j-1}^{l-1}(t).$$

The basis functions used for fitting B-splines in Section 4 of the main manuscript are depicted in Figure S1.

3 On confidence intervals for the time points of interest

In Section 2.2 we obtained time points of significant change in gene expression. However, as estimators, they are subject to uncertainty. Thus, the construction of confidence intervals for these time points is of huge practical importance. The method proposed is an extension to the two-step bootstrap described in Section 2.3. Precisely, another bootstrap level is added in advance, and the two-step procedure described above is then applied to each sample. This provides an estimated lower confidence band for each of the generated data sets, allowing calculation of the time frames of significant change in gene expression and the time points of interest. In the following analysis, only those cases with the same number of coherent subsets as the original data set are considered. The corresponding time points of interest from all bootstrap samples are combined (e.g. all t^*_{start} and all t^*_{end}, where the '*' denotes the bootstrap

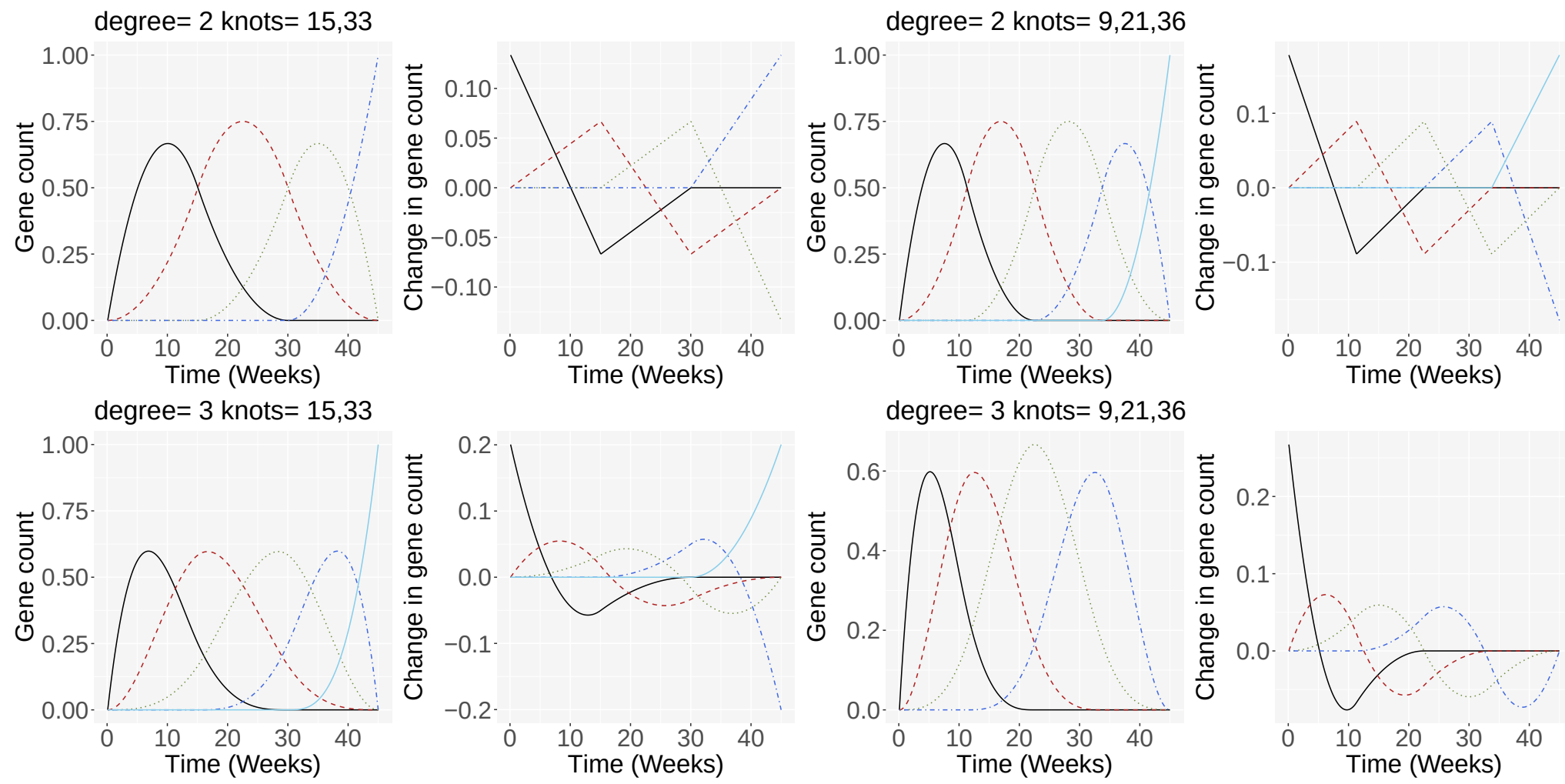

Figure S1: A depiction of the different B-spline basis functions (left) and the respective first derivatives (right) used in Section 4. Of note, as per the default option `intercept=F` of the `R` function *bSpline()* used for the generation of the B-spline basis, the first basis function is left out (Wang, W. and Yan, J., 2021). Therefore, the number of basis functions is $d-1$ and not d as the theoretical approach would indicate. The intercept is included in the estimation via the `R` function *lm()*.

data) and used to estimate the empirical $(\alpha/2)$- and $(1-(\alpha/2))$-quantiles , say $q_{\alpha/2}$ and $q_{(1-(\alpha/2))}$ providing percentile confidence intervals for the time points of interest of the original data set that can be expressed as $[q_{\alpha/2}, q_{(1-(\alpha/2))}]$. Figure B depicts a visualization of this concept, while Algorithm A summarizes the procedure. For the application presented in Section 4, we chose $B_3 = 500$ bootstrap runs. This is consistent with the choice of $B_1 = 500$ for the first-level bootstrap runs, since quantiles are estimated in both cases.

4 Application to an individual gene - detailed analysis

Cd163

'Cd163' is a gene found in both mice (National Center for Biotechnology Information, 2024b) and humans (National Center for Biotechnology Information, 2024a). The soluble form of the encoded molecule was found to be a predictor for both fibrosis and hepatocellular carcinoma development in nonalcoholic steatohepatitis in humans, as reported in Kawanaka, M. and Nishino, K. and Kawada, M. and Ishii, K. and Tanikawa, T. and Katsumata, R. and Urata, N. and Nakamura, J. and Suehiro, M. and Haruma, K. and Kawamoto, H (2023). In particular, decreased levels were observed in patients with NAFLD who exhibited alleviated fibrosis and inflammation. In Ghallab, A. et al. (2021), the gene has been identified as a RJG. This implies that the gene in question is only deregulated after a specific period of time, in this case, after 24 weeks. In a preceding model selection step, the 4pLL model, the exponential model, the

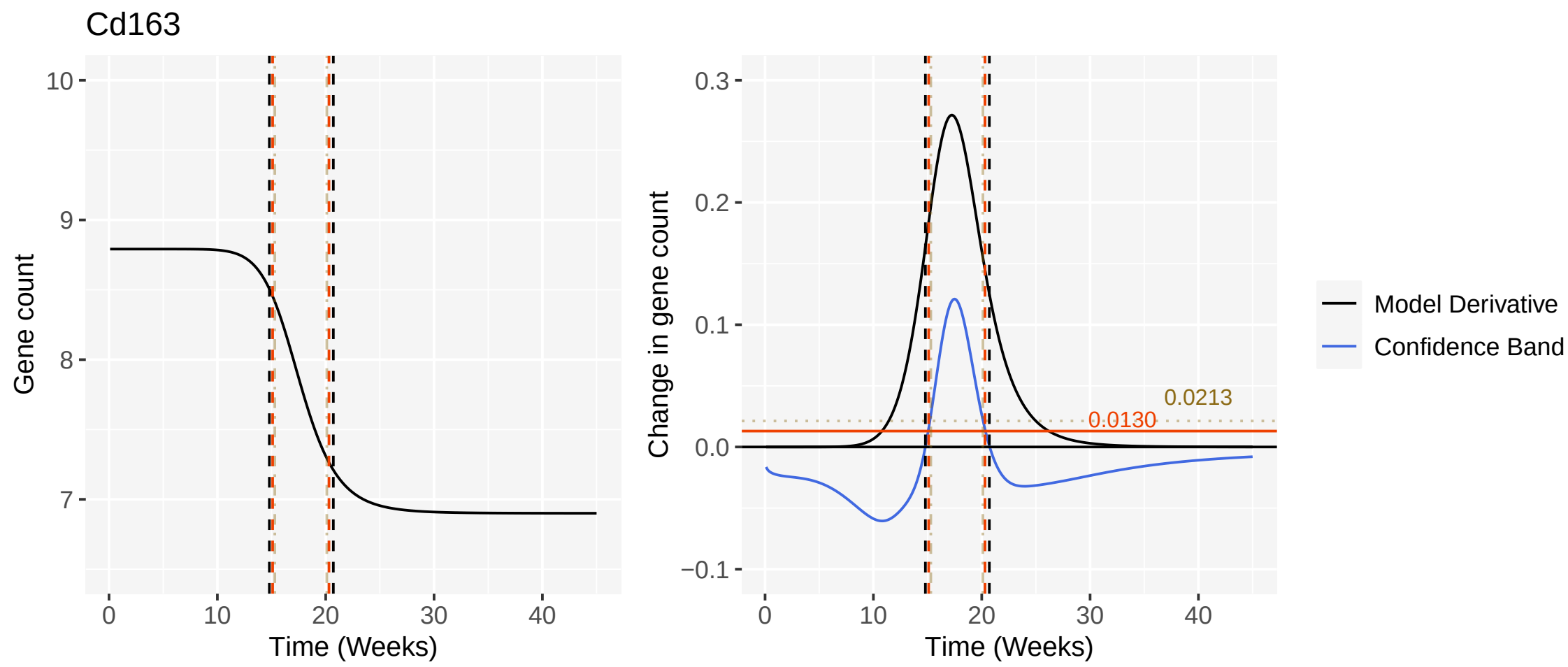

Figure S2: On the left, the fit of the 4pLL model to the normalized gene count of the gene 'Cd163' over time is depicted. On the right, the first derivative and the estimated confidence band of the model fit are displayed. The horizontal red line indicates $\lambda = \log_2(1.5) \times 45^{-1}$. The vertical dashed red lines indicate the corresponding time frame of significant change in gene expression. The yellow colored lines correspond to an alternative choice of $\lambda = \log_2(1.5) \times 27.5^{-1}$. The black lines represent the case $\lambda = 0$.

quadratic model and the beta model were fitted to the gene expression data of the WD-fed mice. The AIC for the 4pLL model was 90.4, whereas for the other models it was between 98.2 and 102.8. Therefore, the 4pLL model was used for further analyses.

The left panel of Figure S2 depicts the estimated gene expression curve. In accordance with the identification as a RJG, it appears that approximately $10 - 12$ weeks pass, before the gene becomes down-regulated. Following an additional $10 - 12$ weeks, the deregulated expression appears to reach a constant value. Overall, the gene changes by a value of -1.891. In the right panel of Figure S2, the corresponding first derivative and the estimated confidence band are depicted. The various horizontal lines represent different potential choices for the threshold λ, while the corresponding vertical lines represent the respective time frames of significant change in gene expression. The black line represents the case where $\lambda = 0$, while the red line corresponds to the aforementioned case $\lambda = \log_2(1.5) \times 45^{-1} = 0.0130$ (see Section 2.2). Similarly, a third threshold was calculated, indicated by the yellow line, which corresponds to a change of $\log_2(1.5)$ in gene expression over the span of half the study. This third threshold is given by $\lambda = \log_2(1.5) \times 27.5^{-1} = 0.0213$.

We observe that H_0 is rejected for all thresholds. In the following, we will present the analysis for $\lambda = \log_2(1.5) \times 45^{-1}$ as an illustrative example. The period during which the estimated significant changes in gene expression occur begins at 15.1 weeks (95% CI $[13.2, 17.8]$) and ends at 20.3 weeks (95% CI $[17.4, 23.5]$). Consequently, we can conclude the classical RJG form of a delayed deregulation. The change is observed over a period of 5.2 weeks, with a plateau following the initial 'jump' in activity. Over the course of the 5.2 week period, the expression

undergoes a change of 1.189. Therefore, we can account for 62.883% of the observed absolute maximal change, 1.891, via a significant effect. Confidence intervals for the time points of interest were estimated using a third-level bootstrap.
For comparison, the other cases indicate a time period of significant change in gene expression between weeks 14.8 to 20.7 ($\lambda = 0$, black) and 15.3 to 20.1 ($\lambda = 0.0213$, yellow), respectively. Consequently, while the 'Cd163' gene was also identified as a RJG, the estimated time frame of deregulation is set earlier than reported in Ghallab, A. et al. (2021) for all thresholds.

As described in Section 4.1. of the main manuscript, the findings derived from fitting B-splines are overall quite similar.

Fam83a

'Fam83a' is a gene found in both mice (National Center for Biotechnology Information, 2025b) and humans (National Center for Biotechnology Information, 2025a), which is associated with the progression of hepatocellular carcinoma (Yu, W.-S. and Wang, Z.-G. and Guo, R.-P. and Lin, Z.-Q. and Ye, Z.-W. and Lu, C.-L., 2020). In Ghallab, A. et al. (2021), the gene has been identified as a RJG that becomes deregulated after 18 weeks. In a preceding model selection step, the 4pLL model, the exponential model, the quadratic model and the beta model were fitted to the gene expression data of the WD-fed mice. The AIC for the beta model was 85.7, whereas for the other models it was between 86.7 and 114.7. Therefore, the beta model was used for further analyses.
The left panel of Figure S3 depicts the estimated gene expression curve. Contrary to the identification as a RJG, it appears that it becomes deregulated immediately. After a period of $20 - 30$ weeks, the deregulated expression seems to attain a constant value. Overall, the gene changes by a value of 2.762. As for the example above, the right panel of Figure S3 depicts the corresponding first derivative, the estimated confidence band and the respective time frame of significant change in gene expression.
We observe that H_0 is rejected for all thresholds. In the following, we will present the analysis for $\lambda = \log_2(1.5) \times 45^{-1}$ as an illustrative example. The period during which the estimated significant changes in gene expression occur begins at 0.3 weeks (95% CI $[0.1, 1.9]$) and ends at 24.9 weeks (95% CI $[15.7, 32.1]$). The change is observed over a period of 24.6 weeks. Over the course of this period, the expression undergoes a change of 2.380. Therefore, we can account for almost all of the observed absolute maximal change via a significant effect.
For comparison, the other cases indicate a time period of significant change in gene expression between weeks 0.3 to 20.7 ($\lambda = 0$, black) and 0.3 to 18.6 ($\lambda = 0.0213$, yellow), respectively. Consequently, while the 'Fam83a' gene was identified as a RJG in Ghallab, A. et al. (2021), the estimated time frame of deregulation begins with the start of the study for all thresholds.

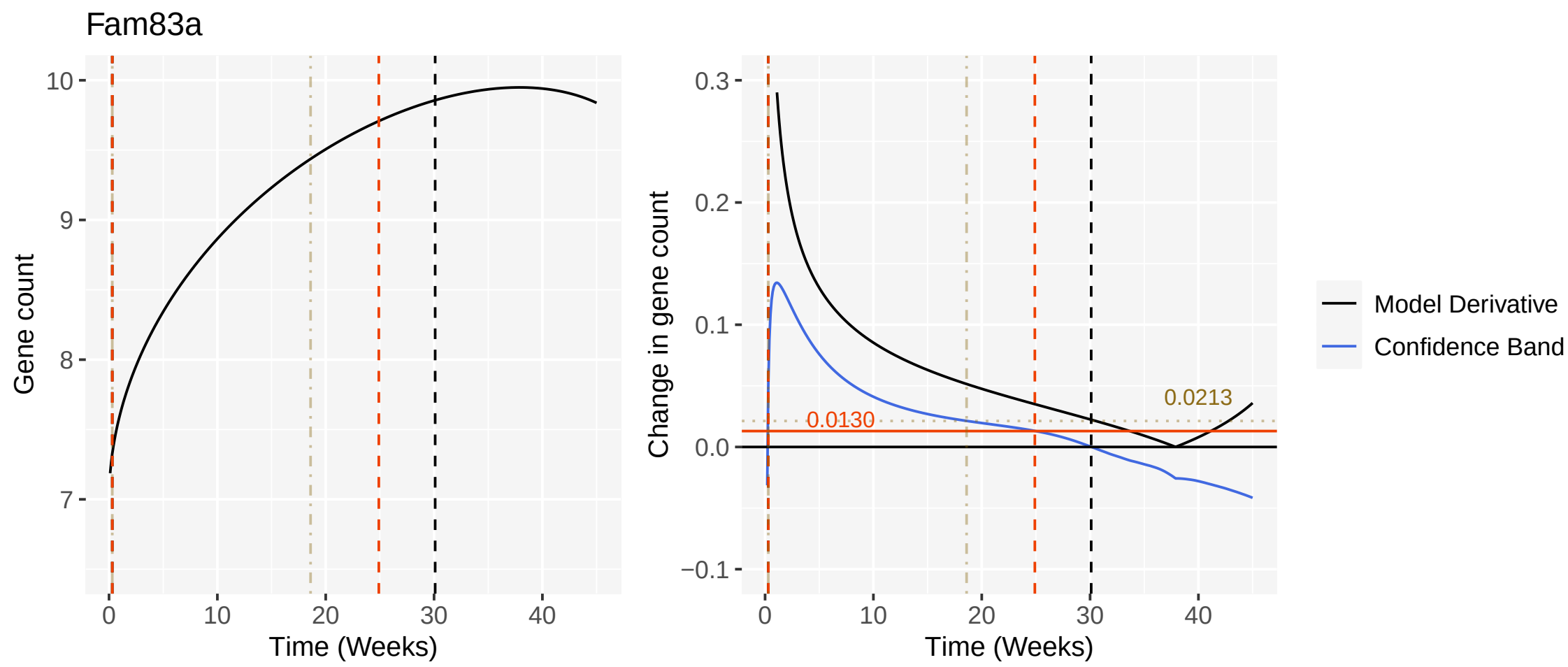

Figure S3: On the left, the fit of the beta model to the normalized gene count of the gene 'Fam83a' over time is depicted. On the right, the first derivative and the estimated confidence band of the model fit are displayed. The horizontal red line indicates $\lambda = \log_2(1.5) \times 45^{-1}$. The vertical dashed red lines indicate the corresponding time frame of significant change in gene expression. The yellow lines correspond to an alternative choice of $\lambda = \log_2(1.5) \times 27.5^{-1}$. The black lines represent the case $\lambda = 0$.

Additionally, an analysis based on fitting B-splines to the data was conducted. The procedure was identical to the one described for the 'Cd163' gene in Section 4.1 of the main manuscript. The results are depicted in Figure S4. Overall, significant change in gene expression was detected from 4 weeks until $17.5 - 22.6$ weeks, which is slightly later but mostly consistent with the results obtained by applying the parametric approach. An exception is observed in the case of $l = 3$ with three knots, wherein the first estimated time period starts with the beginning of the study. However, the fit to the data seems to indicate overfitting. For all cases, except for $l = 2$ degrees and two knots, the time frame of significant change is segmented by an inactive period around week 10. These results are caused by the notable spike in expression at 6 weeks on the study or equivalently a dip of the measurements taken at 9 weeks on the study. This may be a fragment of the data that was incorrectly identified as a period of inactivity due to the flexible nature of B-splines. Nevertheless, it is conceivable that an inactive period exists, which would not be discernible through the application of a comparatively rigid parametric model.

Dbp

'Dbp' is a protein encoding gene detected in humans (National Center for Biotechnology Information, 2024c) and mice (National Center for Biotechnology Information, 2024d) associated with the circadian rhythm. In a preceding model selection step, the 4pLL model, the exponential model, the quadratic model and the beta model were fitted to the gene expression data of the

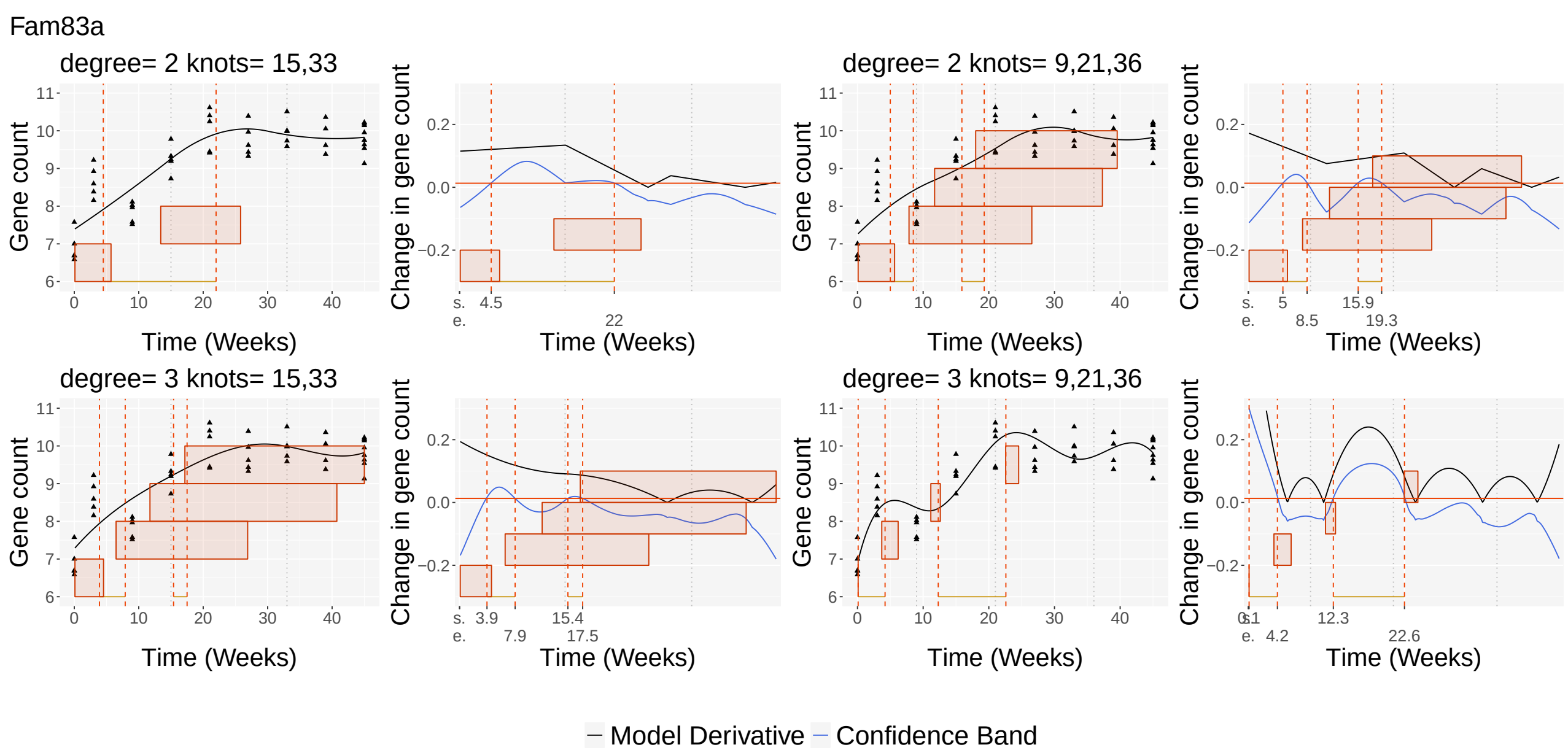

Figure S4: The fit of the B-splines to the normalized gene count of the gene 'Fam83a' over time is depicted on the left for each presented combination of degree and number of knots. The degree of the basis functions and the knot placement are stated in the subtitle of the respective plots. Additionally, the knots are indicated by gray dotted lines. On the right, the first derivatives and the estimated confidence bands are displayed. The horizontal red lines indicate $\lambda = \log_2(1.5) \times 45^{-1}$. The vertical dashed red lines and the horizontal yellow lines indicate the corresponding time frames of significant change in gene expression. The red boxes illustrate the estimated confidence intervals for the respective time points of interest.

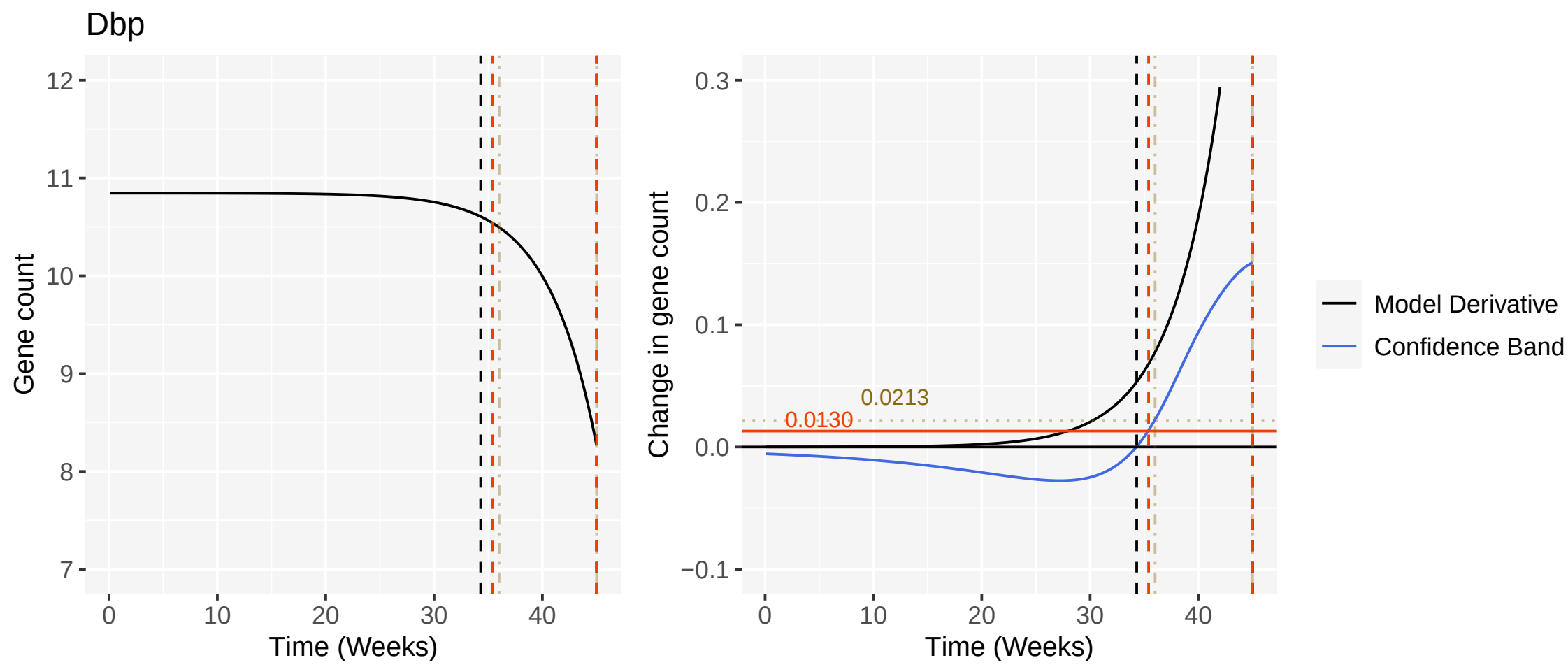

Figure S5: On the left, the fit of the exponential model to the normalized gene count of the gene 'Dbp' over time is depicted. On the right, the first derivative and the estimated confidence band of the model fit are displayed. The horizontal red line indicates $\lambda = \log_2(1.5) \times 45^{-1}$. The vertical dashed red lines indicate the corresponding time frame of significant change in gene expression. The yellow lines correspond to an alternative choice of $\lambda = \log_2(1.5) \times 27.5^{-1}$. The black lines represent the case $\lambda = 0$.

WD-fed mice. The AIC for the exponential model was 141.9, whereas for the other models it was between 143.8 and 148.4. Therefore, the exponential model was used for further analyses. The left panel of Figure S5 depicts the estimated gene expression curve. It appears that it becomes deregulated after approximately 30 weeks. Overall, the gene changes by a value of -2.580. As for the example above, the right panel of Figure S5 depicts the corresponding first derivative, the estimated confidence band and the respective time frames of significant change in gene expression.

We observe that H_0 is rejected for all thresholds. In the following, we will present the analysis for $\lambda = \log_2(1.5) \times 45^{-1}$ as an illustrative example. The period during which the estimated significant changes in gene expression occur begins at 35.4 weeks (95% CI $[29.3, 37.0]$) and appears to continue after the end of the study. The change is observed over a period of 9.6 weeks. Over the course of this period, the expression undergoes a change of 2.275. Therefore, we can account for more than 85% of the observed absolute maximal change, 2.580, via a significant effect.

For comparison, the other cases indicate a time period of significant change in gene expression between weeks 34.3 ($\lambda = 0$, black) and 36.0 ($\lambda = 0.0213$, yellow) till the end of the study, respectively. Consequently, we can identify the 'Dbp' gene as a RJG.

Additionally, an analysis based on fitting B-splines to the data was conducted. The procedure was identical to the one described for the 'Cd163' gene in Section 4.1. of the main manuscript. The results are depicted in Figure S6. Overall, two distinct time frames of significant change

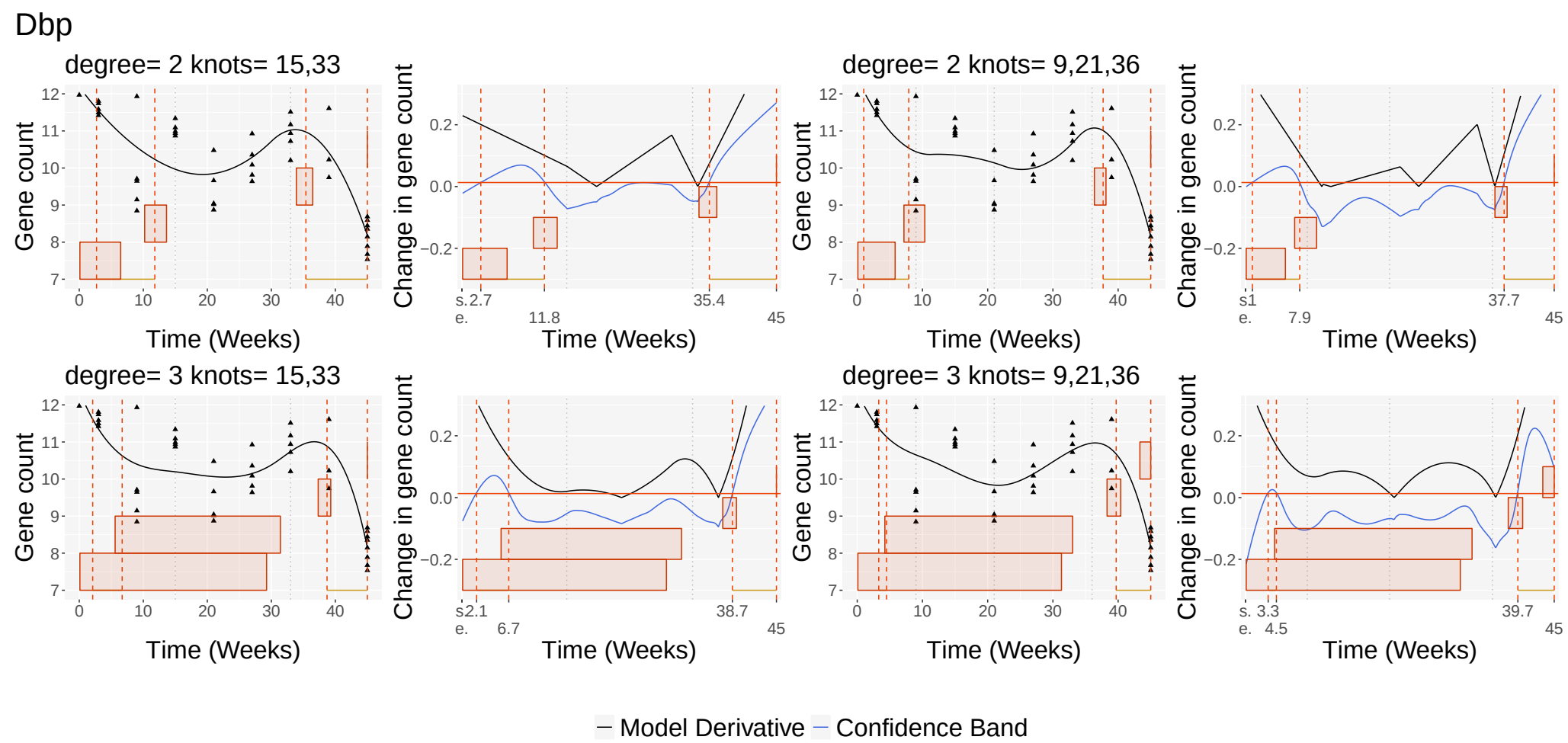

Figure S6: The fit of the B-splines to the normalized gene count of the gene 'Dbp' over time is depicted on the left for each presented combination of degree and number of knots. The degree of the basis functions and the knot placement are stated in the subtitle of the respective plots. Additionally, the knots are indicated by gray dotted lines. On the right, the first derivatives and the estimated confidence bands are displayed. The horizontal red lines indicate $\lambda = \log_2(1.5) \times 45^{-1}$. The vertical dashed red lines and the horizontal yellow lines indicate the corresponding time frames of significant change in gene expression. The red boxes illustrate the estimated confidence intervals for the respective time points of interest.

in expression were detected. The beginning of the later period was detected at $35.5 - 39.7$ weeks up until the end of the study, which is consistent with the results obtained applying the parametric approach. However, the early period of activity was not detected using the parametric model. For $l = 2$ degrees, this period is estimated to last $6.9 - 9.1$ weeks from $1 - 2.7$ weeks until $7.9 - 11.8$ weeks. For $l = 3$ degrees, the period is shorter. It is estimated to start at $2.1 - 3.3$ weeks until $4.5 - 6.7$ weeks. These results demonstrate the benefit of fitting B-splines, since the earlier period of activity was most likely missed by the parametric model due to the predefined shape.

Tm7sf2

'Tm7sf2' is a gene found in both mice (National Center for Biotechnology Information, 2025c) and humans (National Center for Biotechnology Information, 2025d), which is associated with weight gain in mice (Gatticchi, L. and de Las Heras, J.I. and Sivakumar, A. and Zuleger, N. and Roberti, R. and Schirmer, E.C., 2020). In a preceding model selection step, the 4pLL model, the exponential model, the quadratic model and the beta model were fitted to the gene expression data of the WD-fed mice. The AIC for the quadratic model was 32.0, whereas for the other models it was between 32.8 and 40.9. Therefore, the quadratic model was used for further analyses.

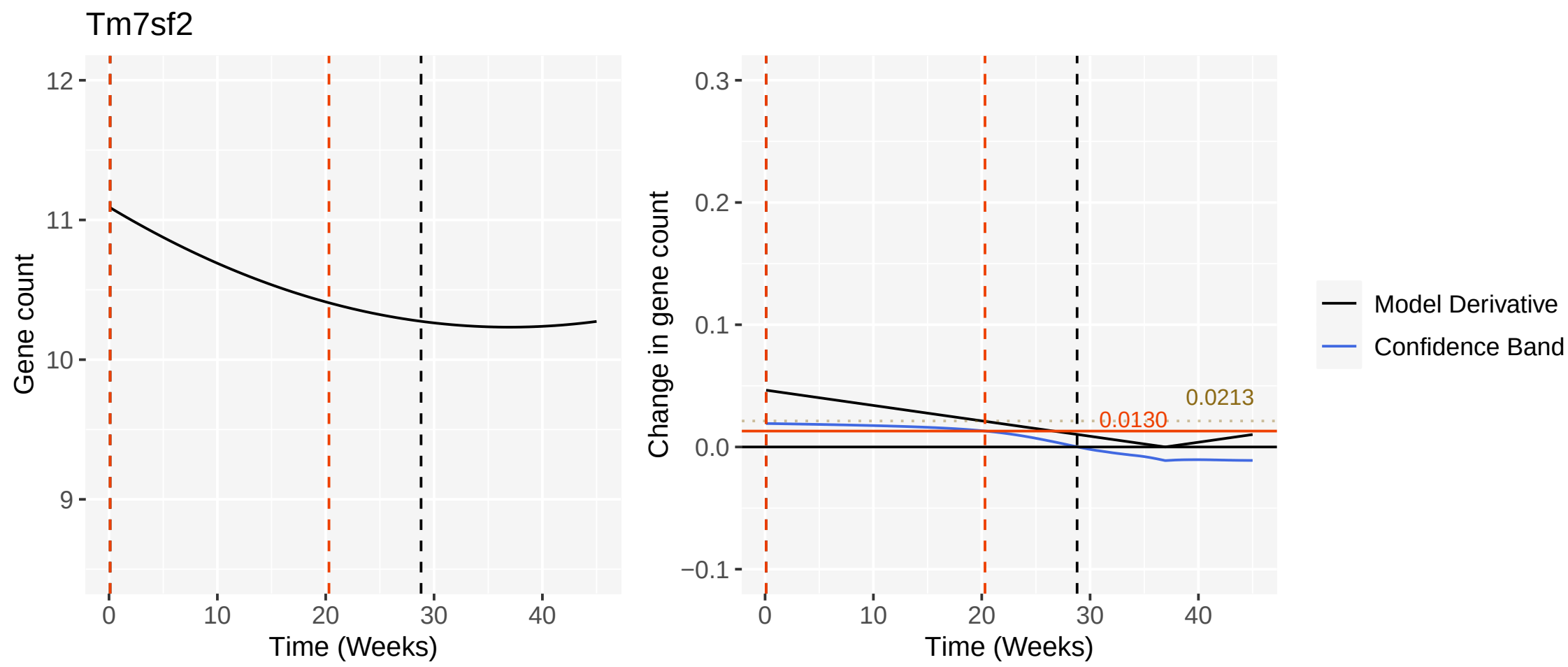

Figure S7: On the left, the fit of the quadratic model to the normalized gene count of the gene 'Tm7sf2' over time is depicted. On the right, the first derivative and the estimated confidence band of the model fit are displayed. The horizontal red line indicates $\lambda = \log_2(1.5) \times 45^{-1}$. The vertical dashed red lines indicate the corresponding time frame of significant change in gene expression. The yellow colored lines correspond to an alternative choice of $\lambda = \log_2(1.5) \times 27.5^{-1}$. The black lines represent the case $\lambda = 0$.

The left panel of Figure S7 depicts the estimated gene expression curve. It appears that it becomes deregulated immediately after the start of the study and reaches a plateau after week 30. Overall the gene changes by a value of -0.855. As for the example above, the right panel of Figure S7 depicts the corresponding first derivative, the estimated confidence band and the respective time frames of significant change in gene expression.
We observe that H_0 is rejected only for the thresholds $\lambda = 0$ and $\lambda = \log_2(1.5) \times 45^{-1}$. H_0 can not be rejected to the α-level 0.05 for $\lambda = \log_2(1.5) \times 27.5^{-1} = 0.0213$. In the following, we will present the analysis for $\lambda = \log_2(1.5) \times 45^{-1}$ as an illustrative example. The period during which the estimated significant changes in gene expression occur begins at the start of the study (95% CI $[0.1, 14.0]$) and end at week 20.3 (95% CI $[12.2, 25.6]$). The change is observed over a period of 20.2 weeks. Over the course of this period, the expression undergoes a change of 0.681. Therefore, we can account for almost 80% of the observed absolute maximal change, 0.855, via a significant effect.
For comparison, the case $\lambda = 0$ indicates a time period of significant change in gene expression between the beginning of the study and week 28.8.

Additionally, an analysis based on fitting B-splines to the data was conducted. The procedure was identical to the one described for the 'Cd163' gene in Section 4.1. of the main manuscript. The results are depicted in Figure S8. Interestingly, there is no significant change in expression detected. We note that the signal that results in the detection of the significant change in expression applying the quadratic model is very small. These results demonstrate the benefit

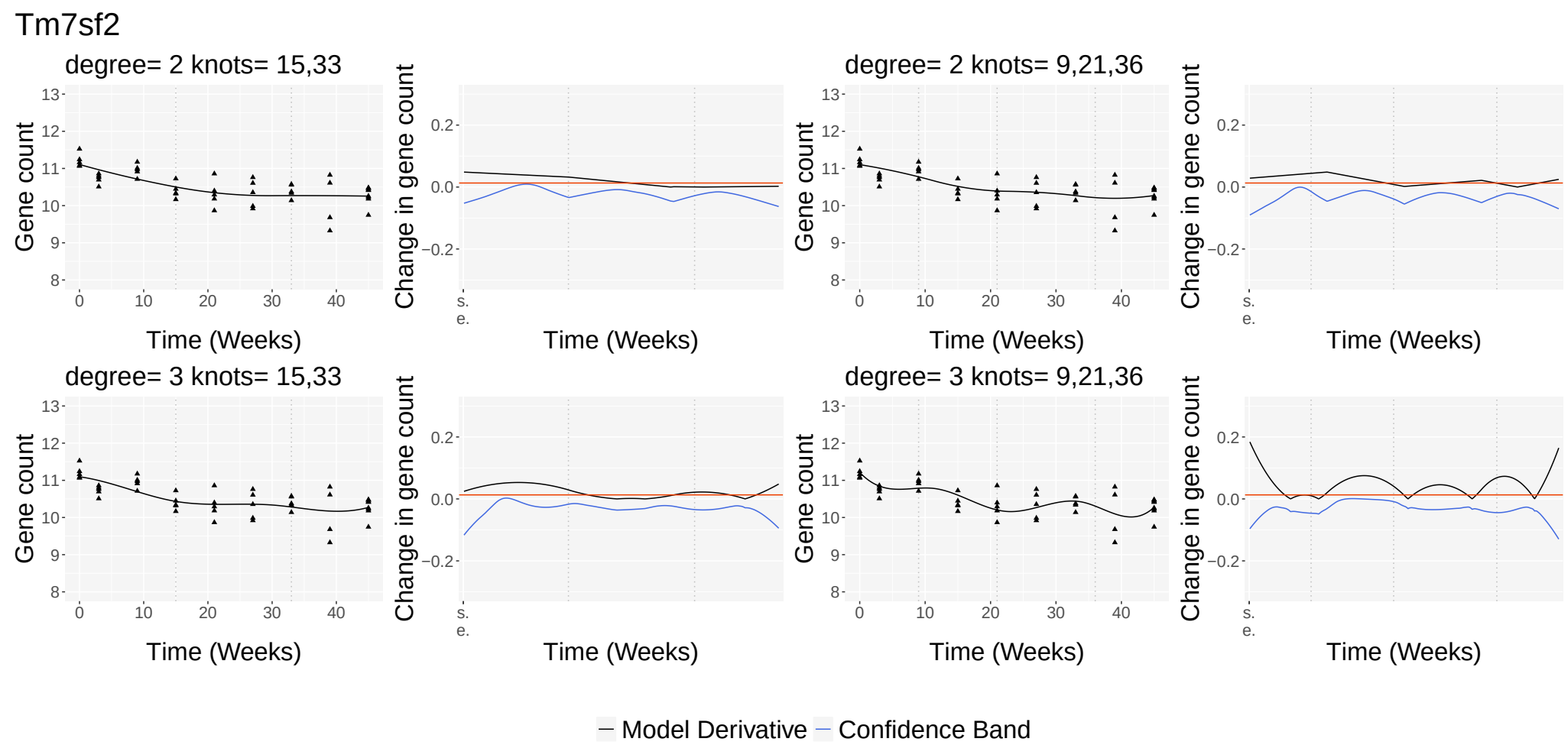

Figure S8: The fit of the B-splines to the normalized gene count of the gene 'Tm7sf2' over time is depicted on the left for each presented combination of degree and number of knots. The degree of the basis functions and the knot placement are stated in the subtitle of the respective plots. Additionally, the knots are indicated by gray dotted lines. On the right, the first derivatives and the estimated confidence bands are displayed. The horizontal red lines indicates $\lambda = \log_2(1.5) \times 45^{-1}$.

of fitting a parametric model, since the procedure, given a well specified model, appears to be more sensitive.

5 Comparison of two groups

For this application we consider a gene that deregulates in both diet groups and compare the estimated time frames of significant change in gene expression. The biological question inspiring this example is whether the WD accelerates or decelerates, and thereby 'ages' biological processes, which also occur in SD-fed mice. The chosen example, 'Dbp', is a protein encoding gene detected in humans (National Center for Biotechnology Information, 2024c) and mice (National Center for Biotechnology Information, 2024d) associated with the circadian rhythm. A preliminary model selection step indicated a superior fit of the exponential model to the gene expression data for both diet groups. Consequently, it was utilised in the subsequent analysis.

The left panel of Figure S9 depicts the modeled gene expression of both the SD and the WD groups. The gene count in SD-fed mice initially appears to be smaller and both cases seem to undergo similar deregulation at approximately the same time and converge to the same value. On the right, the corresponding first derivatives and the estimated confidence bands are displayed. The dotted line represents the time period of significant change in gene expression for the WD-fed group, while the dot-dashed line outlines the time period for the SD-fed mice. Although the first derivative of the WD-fed group appears to increase earlier, the confidence

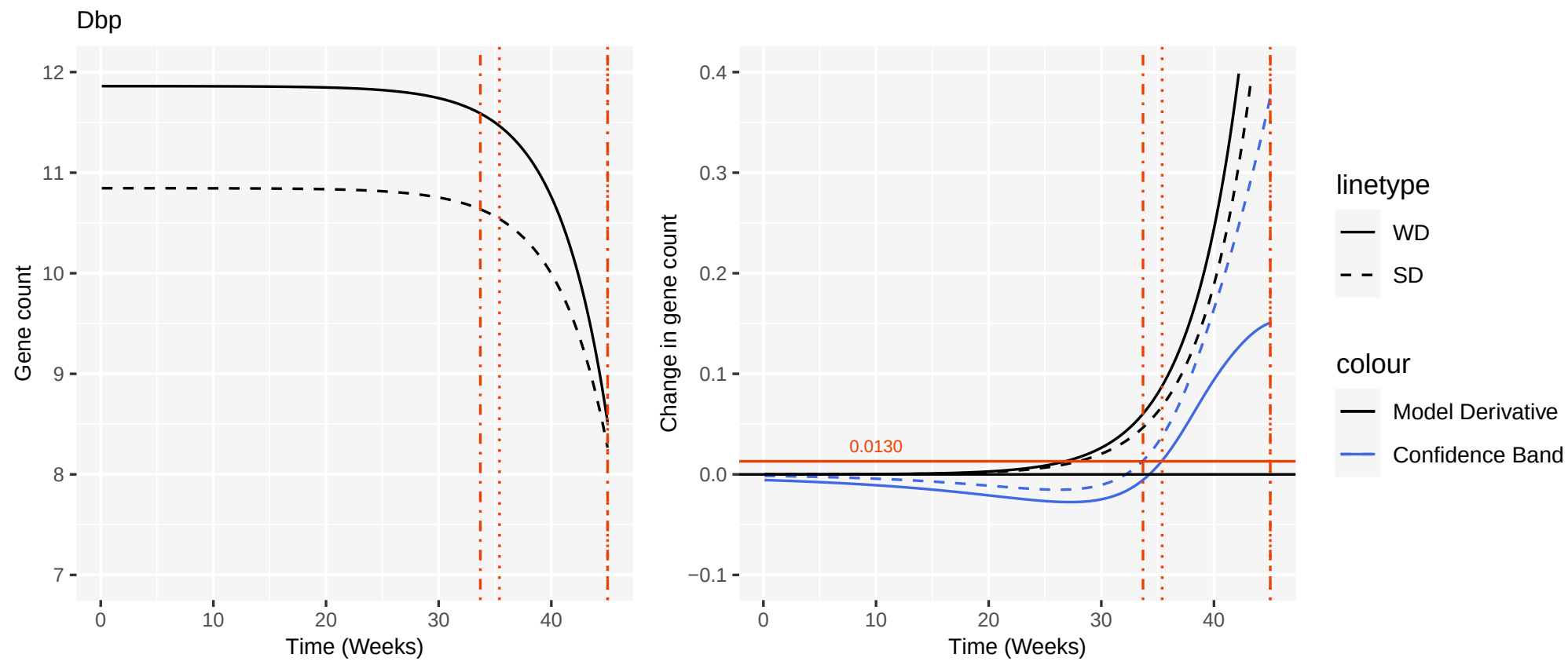

Figure S9: A comparison of the SD versus the WD, exemplary for the gene 'Dbp'. The gene counts, as modeled by the exponential model, are depicted on the left. On the right, the first derivatives and their estimated confidence bands are shown. The red dotted line outlines the time period of significant change in gene expression for the WD, while the dot-dashed line outlines the analogous period for the SD.

band of the SD group exceeds the threshold $\lambda = \log_2(1.5) \times 45^{-1} = 0.0130$ earlier. For a detailed discussion of the results for the WD-fed mice see the section above. The estimated time period during which significant change in gene expression in SD-fed mice occurs begins at 33.7 weeks (95% CI $[28.8, 34.4]$) and ends with the end of study at 45 weeks (95% CI $[45.0, 45.0]$, for computational reasons). The gene count undergoes a change of 3.070 over the span of 11.3 weeks. Overall, both genes show very similar results. The estimated confidence band for the SD-fed mice exceeds the threshold $\lambda = 0.0130$ about -1.7 weeks earlier and the estimated 95% confidence interval for this difference is given by $[-6.4, 4.1]$.

It should be noted that no measurements were taken from SD-fed mice between weeks 3 and 27. Hence, when interpreting the results of a comparison of the two diets during this time period, this should be taken into account. Since the parametric fit indicates activity only after week 30, this is not a problem for the results presented here. However, due to their flexible nature, this causes problems when applying the method using B-splines. Therefore, we limit the proof of concept presented here to the parametric approach.

References

Bornkamp, B. and Pinheiro, J. and Bretz, F. MCPMod: An R Package for the Design and Analysis of Dose-Finding Studies. *Journal of Statistical Software*, 29(7):1 – 23, 2009. doi: 10.18637/jss.v029.i07.

Bornkamp, B. and Pinheiro, J. and Bretz, F. *MCPMod: An R Package for the Design and*

Analysis of Dose-Finding Studies, 2017. URL https://CRAN.R-project.org/package=MCPMod. R package version 1.0-10.1.

Fahrmeir, L. and Kneib, T. and Lang, S. and Marx, B.D. *Regression: Models, Methods and Applications*. Springer Berlin, Heidelberg, 2nd edition, 2022. doi: 10.1007/978-3-662-63882-8.

Gatticchi, L. and de Las Heras, J.I. and Sivakumar, A. and Zuleger, N. and Roberti, R. and Schirmer, E.C. Tm7sf2 disruption alters radial gene positioning in mouse liver leading to metabolic defects and diabetes characteristics. *Frontiers in Cell and Developmental Biology*, 8:592573, 2020. doi: 10.3389/fcell.2020.592573.

Ghallab, A., Myllys, M., Friebel, A., Duda, J., Edlund, K., Halilbasic, E., Vucur, M., Hobloss, Z., Brackhagen, L., Begher-Tibbe, B., Hassan, R., Burke, M., Genc, E., Frohwein, L.J., Hofmann, U., Holland, C.H., González, D., Keller, M., Seddek, A., Abbas, T., Mohammed, E.S.I., Teufel, A., Itzel, T., Metzler, S., Marchan, R., Cadenas, C., Watzl, C., Nitsche, M.A., Kappenberg, F., Luedde, T., Longerich, T., Rahnenführer, J., Hoehme, S., Trauner, M., and Hengstler, J.G. Spatio-Temporal Multiscale Analysis of Western Diet-Fed Mice Reveals a Translationally Relevant Sequence of Events during NAFLD Progression. *Cells*, 10(10), 2021. doi: 10.3390/cells10102516.

Kawanaka, M. and Nishino, K. and Kawada, M. and Ishii, K. and Tanikawa, T. and Katsumata, R. and Urata, N. and Nakamura, J. and Suehiro, M. and Haruma, K. and Kawamoto, H. Soluble CD163 is a predictor of fibrosis and hepatocellular carcinoma development in nonalcoholic steatohepatitis. *BMC gastroenterology*, 23(1), 2023. doi: 10.1186/s12876-023-02786-4.

National Center for Biotechnology Information. *CD163 molecule [Homo sapiens (human)]*. 2024a. URL https://www.ncbi.nlm.nih.gov/gene/93671. Accessed June 13, 2024.

National Center for Biotechnology Information. *Cd163 antigen [Mus musculus (house mouse)]*. 2024b. URL https://www.ncbi.nlm.nih.gov/gene/93671. Accessed June 13, 2024.

National Center for Biotechnology Information. *DBP D-box binding PAR bZIP transcription factor [Homo sapiens (human)]*. 2024c. URL https://www.ncbi.nlm.nih.gov/gene/1628. Accessed June 14, 2024.

National Center for Biotechnology Information. *Dbp D site albumin promoter binding protein [Mus musculus (house mouse)]*. 2024d. URL https://www.ncbi.nlm.nih.gov/gene/13170. Accessed June 14, 2024.

National Center for Biotechnology Information. *FAM83A family with sequence similarity 83 member A [Homo sapiens (human)]*. 2025a. URL https://www.ncbi.nlm.nih.gov/gene/84985. Accessed May 16, 2025.

National Center for Biotechnology Information. *Fam83a family with sequence similarity 83, member A [Mus musculus (house mouse)]*. 2025b. URL https://www.ncbi.nlm.nih.gov/gene/239463. Accessed May 16, 2025.

National Center for Biotechnology Information. *Tm7sf2 transmembrane 7 superfamily member 2 [Mus musculus (house mouse)]*. 2025c. URL https://www.ncbi.nlm.nih.gov/gene/73166. Accessed May 16, 2025.

National Center for Biotechnology Information. *TM7SF2 transmembrane 7 superfamily member 2 [Homo sapiens (human)]*. 2025d. URL https://www.ncbi.nlm.nih.gov/gene/7108. Accessed May 16, 2025.

Wang, W. and Yan, J. Shape-restricted regression splines with R package splines2. *Journal of Data Science*, 19(3):498–517, 2021. doi: 10.6339/21-JDS1020.

Yu, W.-S. and Wang, Z.-G. and Guo, R.-P. and Lin, Z.-Q. and Ye, Z.-W. and Lu, C.-L. Hepatocellular carcinoma progression is protected by miRNA-34c-5p by regulating FAM83A. *European Review for Medical & Pharmacological Sciences*, 24(11), 2020. doi: 10.26355/eurrev_202006_21499.

Table S1: Levels of standard deviation used for the simulation study. The standard deviations marked with an asterisk (*) are approximately half the size of the maximum change in gene expression.

Sce.	small	mid-small	medium	mid-large	large
1	0.014	0.021	0.028	0.042	0.056*
2	0.149	0.223	0.297	0.446	0.595*
3	0.595	0.892	1.190	1.784*	2.380*
4	0.283	0.424	0.566	0.849	1.132
5	0.283	0.424	0.566	0.849	1.132
6	0.283	0.424	0.566	0.849	1.132

Table S2: Simulation results. 'CorRuns' indicates the number of simulation runs, for which the correct number of coherent subsets of $\tilde{S}$ were detected. The suffix '_s1/2' indicates the start of $S_{1/2}$ and correspondingly '_e1' the end. Since S_2 ends at the end of study, it is not considered here.

Sci.	σ	CorRuns	Bias_s1	Var_s1	Bias_e1	Var_e1	Bias_s2	Var_s2
Sce. 2	small σ	998	2.339	0.584	-2.819	0.917	-	-
Sce. 2	mid-small σ	935	3.323	1.319	-3.974	1.896	-	-
Sce. 2	medium σ	828	3.877	1.849	-4.745	2.424	-	-
Sce. 2	mid-large σ	625	4.823	2.777	-5.16	3.893	-	-
Sce. 2	large σ	371	5.413	6.359	-5.24	8.592	-	-
Sce. 3	small σ	989	5.398	2.93	-10.135	8.996	-	-
Sce. 3	mid-small σ	617	7.056	7.206	-13.21	20.138	-	-
Sce. 3	medium σ	442	8.431	15.564	-14.038	35.942	-	-
Sce. 3	mid-large σ	392	10.421	10.989	-16.256	16.88	-	-
Sce. 3	large σ	301	11.369	10.746	-16.347	16.157	-	-
Sce. 4	small σ	118	0.005	0.001	-3.951	1.85	2.631	1.001
Sce. 4	mid-small σ	44	0.12	0.046	-5.963	5.435	2.4	1.627
Sce. 4	medium σ	43	0.358	0.285	-8.897	17.891	2.188	2.042
Sce. 4	mid-large σ	33	1.27	3.535	-16.938	34.394	2.076	2.706
Sce. 4	large σ	37	2.379	10.036	-21.142	35.308	1.882	3.45
Sce. 5	small σ	999	3.956	0.981	-1.225	0.236	2.074	1.065
Sce. 5	mid-small σ	864	5.267	1.375	-1.845	0.567	2.912	2.053
Sce. 5	medium σ	671	6.244	1.912	-2.535	1.174	3.141	1.999
Sce. 5	mid-large σ	424	7.5	4.88	-4.932	11.235	3.475	1.909
Sce. 5	large σ	260	8.865	11.986	-7.61	27.344	3.339	12.426
Sce. 6	small σ	993	0	0	-6.996	7.686	-	-
Sce. 6	mid-small σ	992	0	0	-11.503	22.59	-	-
Sce. 6	medium σ	995	0.003	0.001	-16.461	33.71	-	-
Sce. 6	mid-large σ	964	0.103	0.153	-21.628	27.636	-	-
Sce. 6	large σ	749	0.375	1.405	-23.591	21.108	-	-

Table S3: Top 20 results of the GO analysis performed on the selected 569 genes. An. for Annotated; Sign. for Significant; Exp. for Expected

Term	An.	Sign.	Exp.	go_pval_adj
alpha-amino acid catabolic process	57	16	3.33	0.0005
surfactant homeostasis	13	8	0.76	0.0005
positive regulation of phagocytosis	70	17	4.09	0.0009
phagocytosis	175	36	10.23	0.0025
positive regulation of tumor necrosis fa...	97	19	5.67	0.0035
branched-chain amino acid catabolic proc...	13	7	0.76	0.0035
negative regulation of interleukin-6 pro...	39	11	2.28	0.0099
negative regulation of IP-10 production	7	5	0.41	0.0106
xenobiotic metabolic process	72	15	4.21	0.0106
positive regulation of interleukin-6 pro...	81	16	4.73	0.0106
bile acid biosynthetic process	23	8	1.34	0.0199
negative regulation of cytokine producti...	18	7	1.05	0.0252
regulation of B cell differentiation	32	9	1.87	0.0361
regulation of ribonuclease activity	9	5	0.53	0.0373
amino acid catabolic process	70	19	4.09	0.0383
positive regulation of interleukin-10 pr...	33	9	1.93	0.0383
inflammatory response	525	64	30.68	0.0445
positive regulation of adaptive immune r...	91	18	5.32	0.0478
mast cell activation	55	14	3.21	0.0478
immunoglobulin mediated immune response	133	23	7.77	0.0492

Algorithm A. *Let $\hat{\boldsymbol{\theta}}$, $\hat{\boldsymbol{\Sigma}}$, $\tilde{S}$ and the time points of interest be calculated from the original data set as described in Section 2. In the event of independent measurements, it is possible to replace $\hat{\boldsymbol{\Sigma}}$ with $\hat{\sigma}^2$, as outlined in Section 3.*

Step 1 *Generate bootstrap data at the m different time points using $\hat{\boldsymbol{\theta}}$ and $\hat{\boldsymbol{\Sigma}}$. Do so by drawing iid. error terms $\boldsymbol{\varepsilon}_p^* = \big(\varepsilon_{p,1}^*, \ldots, \varepsilon_{p,m}^*\big)^* \sim N_m\big(\mathbf{0}, \hat{\boldsymbol{\Sigma}}\big)$ and applying the formula*

$$y_{p,q}^* = f\big(t_q, \hat{\boldsymbol{\theta}}\big) + \varepsilon_{p,q}^* \text{ for } p = 1, \ldots, n_q,\ q = 1, \ldots, m.$$

If the measurements are independent: Draw iid. errors $\varepsilon_{p,q}^* \sim N\left(0, \hat{\sigma}^2\right)$.

Repeat this step B_3 times. This returns $y_{p,q}^{*,1}, \ldots, y_{p,q}^{*,B_3}$.

Next, calculate the estimates $\hat{\boldsymbol{\theta}}_k^*$ and $\hat{\boldsymbol{\Sigma}}_k^*$ (or $\hat{\sigma}_k^{2,*}$), $k = 1, \ldots, B_3$ as descried in Section 2.1. for each of the generated data sets. This yields $\big|f'\big(t, \hat{\boldsymbol{\theta}}_k^*\big)\big|$.

Now for $k = 1, \ldots, B_3$ perform the two-step bootstrap procedure to estimate the confidence band:

Step 2 *For $k = 1, \ldots, B_3$: Generate bootstrap data at the m different time points using $\hat{\boldsymbol{\theta}}_k^*$ and $\hat{\boldsymbol{\Sigma}}_k^*$. Do so by drawing iid. error terms $\boldsymbol{\varepsilon}_p^{**} = \big(\varepsilon_{p,1}^{**}, \ldots, \varepsilon_{p,m}^{**}\big) \sim N_m\big(\mathbf{0}, \hat{\boldsymbol{\Sigma}}_k^*\big)$ and applying the formula*

$$y_{p,q}^{**} = f\big(t_q, \hat{\boldsymbol{\theta}}_k^*\big) + \varepsilon_{p,q}^{**} \text{ for } p = 1, \ldots, n_q,\ q = 1, \ldots, m.$$

If the measurements are interdependent Draw iid. errors $\varepsilon_{p,q}^{**} \sim N\big(0, \hat{\sigma}_k^{2,*}\big)$

Repeat this step B_1 times. This returns $y_{p,q}^{**,1}, \ldots, y_{p,q}^{**,B_1}$.

For each combination of $k = 1, \ldots, B_3$ and $l = 1, \ldots, B_1$ estimate $\hat{\boldsymbol{\theta}}_{k,l}^{**}$ and $\hat{\boldsymbol{\Sigma}}_{k,l}^{**}$ (or $\hat{\sigma}_{k,l}^{2,**}$). This yields $\big|f'\big(t, \hat{\boldsymbol{\theta}}_{k,l}^{**}\big)\big|$.

The replicates $\big|f'\big(t, \hat{\boldsymbol{\theta}}_{k,1}^{**}\big)\big|, \ldots, \big|f'\big(t, \hat{\boldsymbol{\theta}}_{k,B_1}^{**}\big)\big|$ can be interpreted as the second level of the bootstrap. The standard error of this sample yield the estimate $\hat{\sigma}_{|f'(t,\hat{\boldsymbol{\theta}}_k^*)|}$.

Step 3 *For each combination of $k = 1, \ldots, B_3$ and $l = 1, \ldots, B_1$: Generate bootstrap data at the m different time points using $\hat{\boldsymbol{\theta}}_{k,l}^{**}$ and $\hat{\boldsymbol{\Sigma}}_{k,l}^{**}$. Do so by drawing iid. error terms $\boldsymbol{\varepsilon}_p^{***} = \big(\varepsilon_{p,1}^{***}, \ldots, \varepsilon_{p,m}^{***}\big) \sim N_m\big(\mathbf{0}, \hat{\boldsymbol{\Sigma}}_{k,l}^{**}\big)$ and applying the formula*

$$y_{p,q}^{***} = f\big(t_q, \hat{\boldsymbol{\theta}}_{k,l}^{**}\big) + \varepsilon_{p,q}^{***} \text{for } p = 1, \ldots, n_q,\ q = 1, \ldots, m.$$

If the measurements are independent: Draw iid. errors $\varepsilon_{p,q}^{***} \sim N\big(0, \hat{\sigma}_{k,l}^{2,**}\big)$

Repeat this step B_2 times.

Estimate $\hat{\boldsymbol{\theta}}^{***}$ (indices omitted). This yields B_2 replicates of the form $\big|f'\big(t, \hat{\boldsymbol{\theta}}^{***}\big)\big|$. The standard errors of the B_2 replicates yield estimates $\hat{\sigma}_{|f'(t,\hat{\boldsymbol{\theta}}_{k,l}^{**})|}$.

Step 4 *For each combination of* $k = 1, \ldots, B_3$ *and* $l = 1, \ldots, B_1$*: Calculate*

$$D^{*,k,l} := \max_{t \in T} \frac{\left|f'\big(t, \hat{\boldsymbol{\theta}}^{**}_{k,l}\big)\right| - \left|f'\big(t, \hat{\boldsymbol{\theta}}^{*}_{k}\big)\right|}{\hat{\sigma}_{|f'(t, \hat{\boldsymbol{\theta}}^{**}_{k,l})|}}.$$

The respective empiric $(1-\alpha)$*-quantiles of the distribution yield estimates of the critical values* c^*_k*.*

Step 5 *For* $k = 1, \ldots, B_3$*: Calculate the lower simultaneous confidence band*

$$L^{\alpha}\big(t, \hat{\boldsymbol{\theta}}^{*}_{k}\big) = \left|f'\big(t, \hat{\boldsymbol{\theta}}^{*}_{k}\big)\right| + c^*_k \hat{\sigma}_{|f'(t, \hat{\boldsymbol{\theta}}^{*}_{k})|}.$$

Calculate the time frames of significant change in gene expression:

$$\tilde{S}_k := \Big\{ t : L^{\alpha}\big(t, \hat{\boldsymbol{\theta}}^{*}_{k}\big) > \lambda \Big\}$$

Partition the $\tilde{S}_k$ *into coherent subsets:*

$$\tilde{S}_k = (S_{k,1}, \ldots, S_{k,s})$$

Step 6 *For* $k = 1, \ldots, B_3$*: Dismiss the cases with more or less coherent subsets then the original data set. Identify the time points of interest for the remaining runs. Now, calculate the empiric* $(\alpha/2)$*- and* $(1-(\alpha/2))$*-quantiles (*$Q_{\alpha/2}$ *and* $Q_{(1-\alpha/2)}$*) for each time point of interest, respectively. The resulting confidence intervals are:*

$$\Big[Q_{\frac{\alpha}{2}}, Q_{(1-\frac{\alpha}{2})}\Big]$$

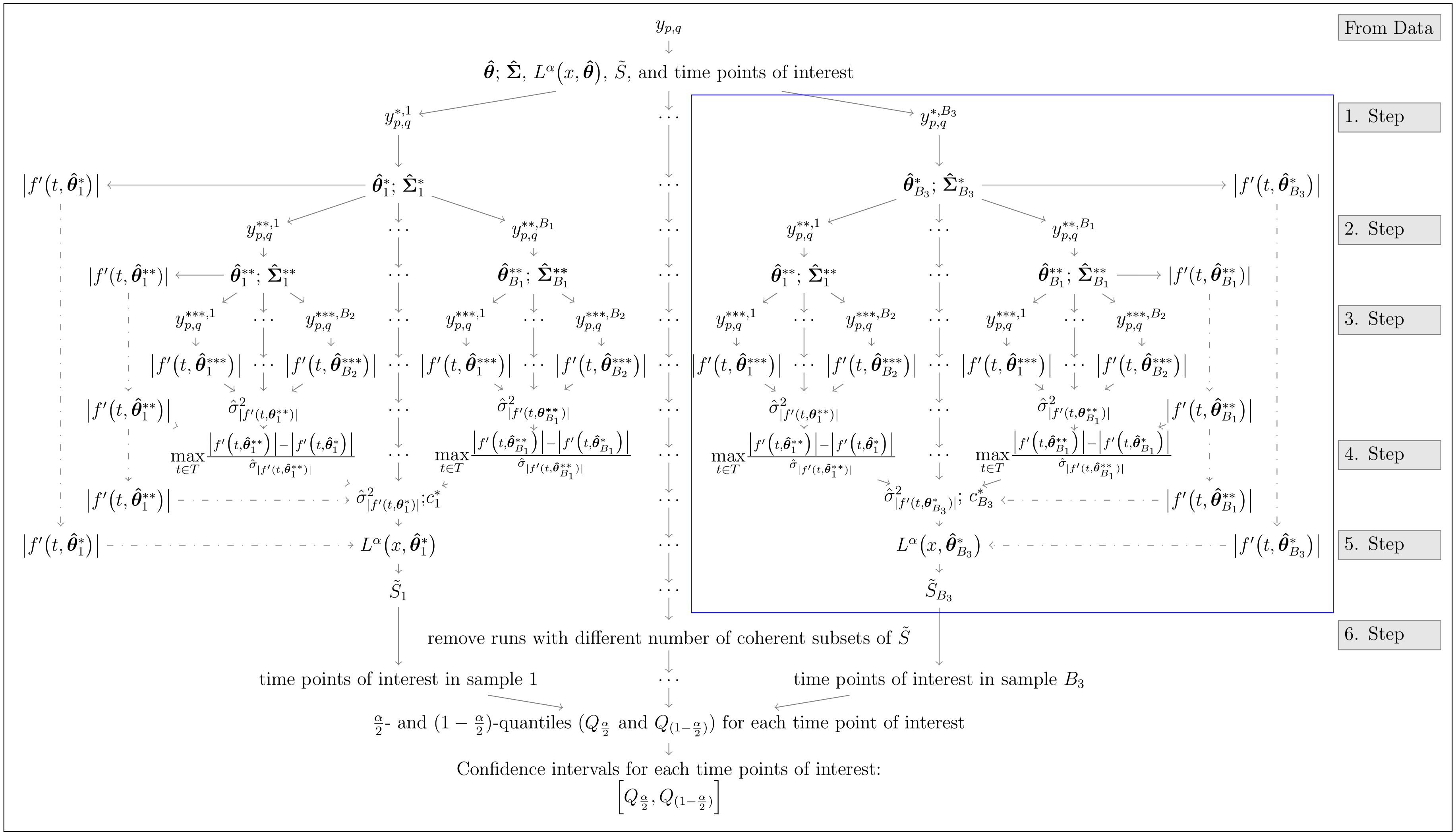

Figure S10: Flowchart of the algorithm described in Subsection 2.4. The blue rectangle indicates the procedure described in Section 2.3. If all measurements are independent the assumption of independent multivariate normal distributed error vectors can be simplified to assuming independent normally distributed errors and $\hat{\boldsymbol\Sigma}^{\,,*,**,***}$ is replaced by $\hat\sigma^{2,\,,*,**,***}$ as described in Section 3.1.

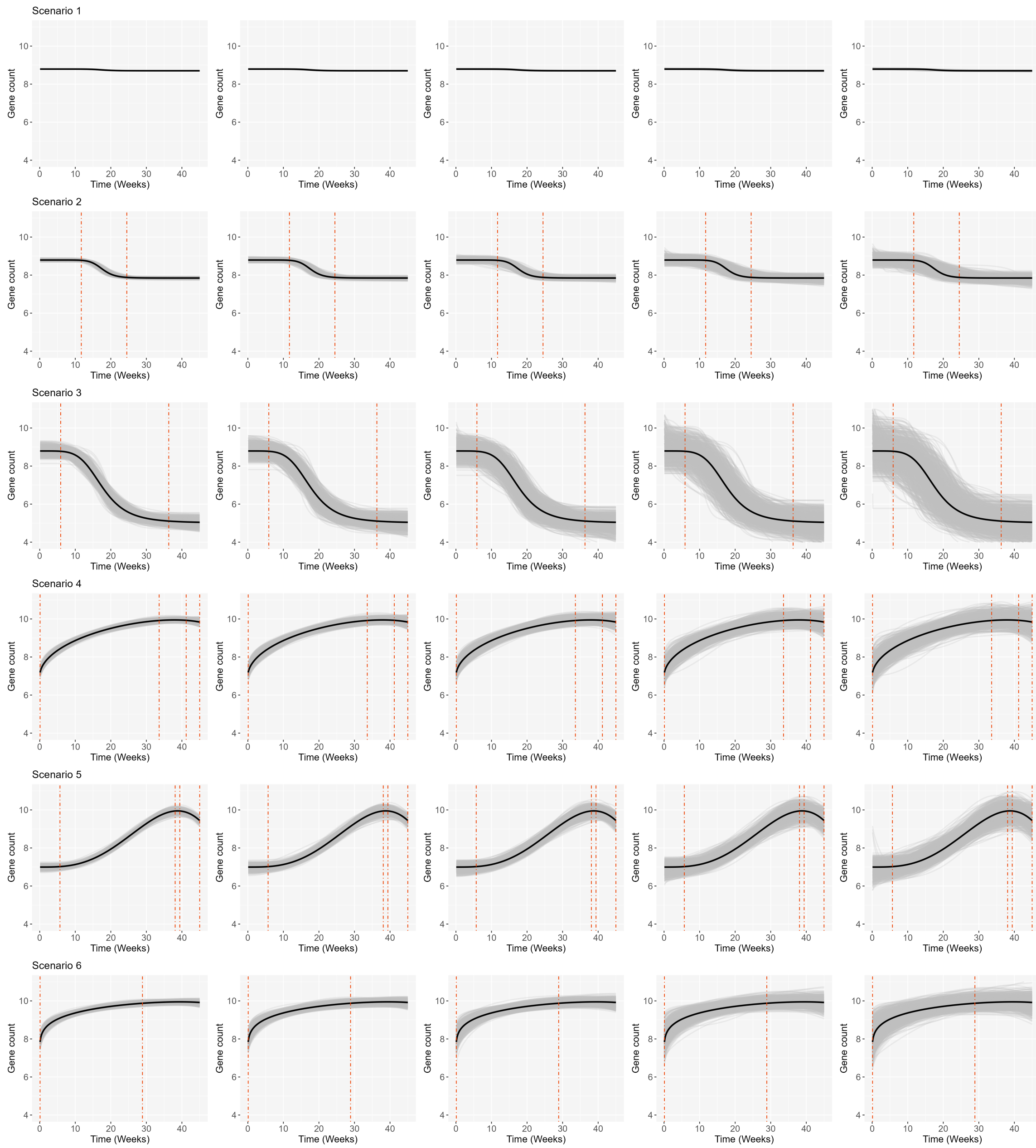

Figure S11: Comparison of the true model function with the estimated model functions derived from each of the simulation studies. The standard deviation increases from left to right.

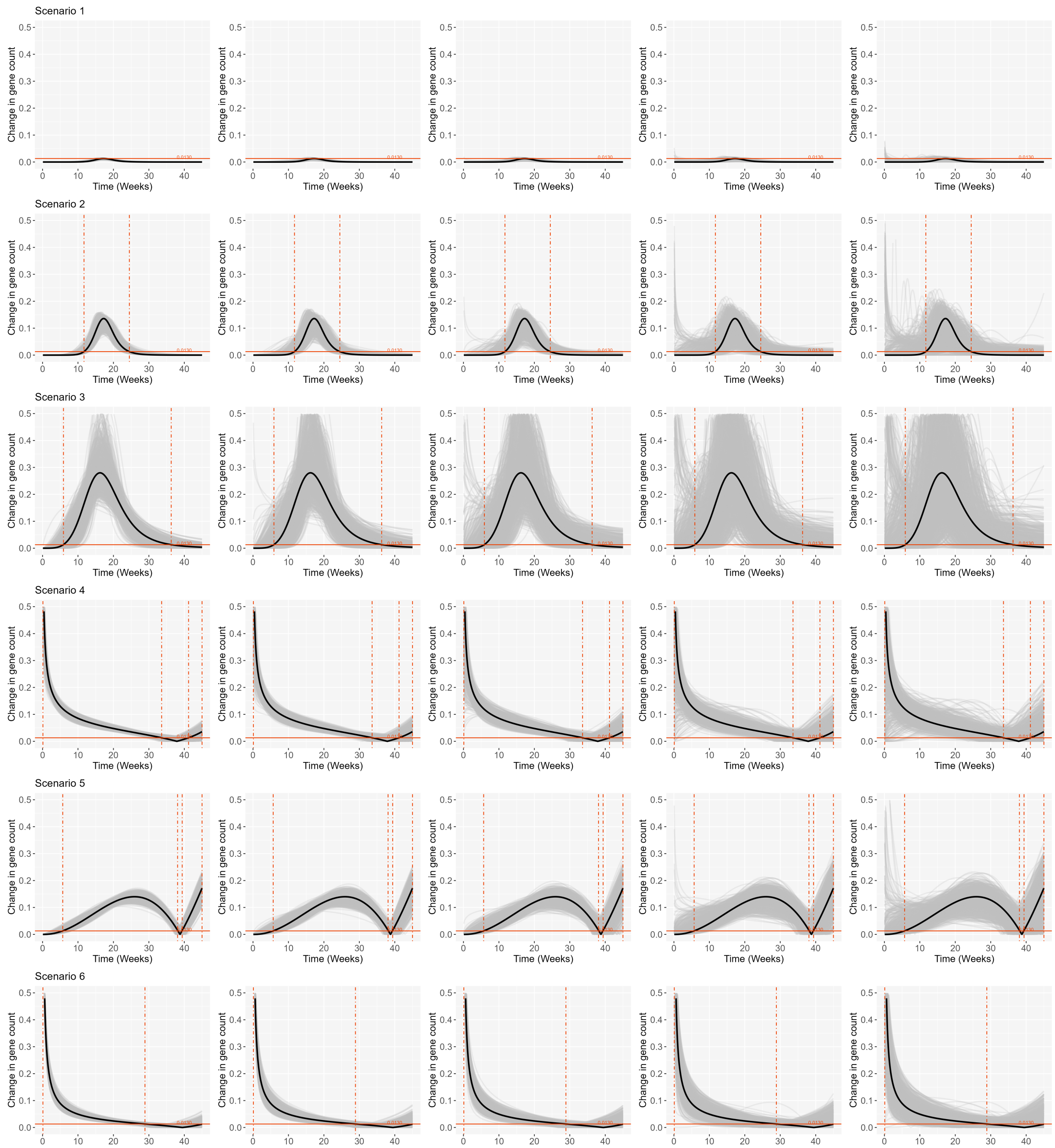

Figure S12: Comparison of the true first derivative with the estimated first derivatives derived from each of the simulation studies. The standard deviation increases from left to right.

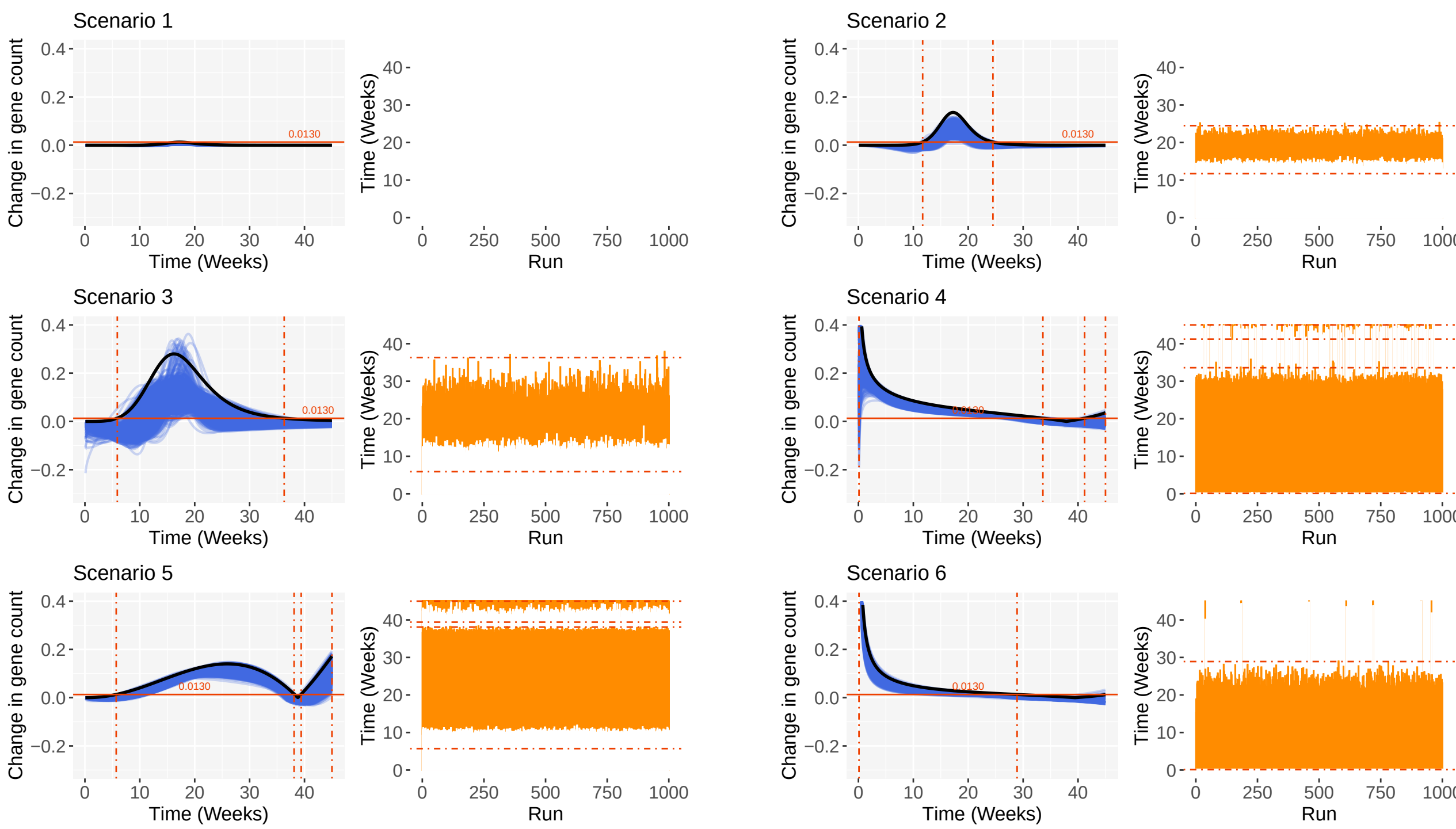

Figure S13: Results of the six simulation scenarios for a small standard deviation. The left column depicts the true first derivative and the estimated confidence bands. The right column depicts the estimated time frames of significant change in gene expression for each run.

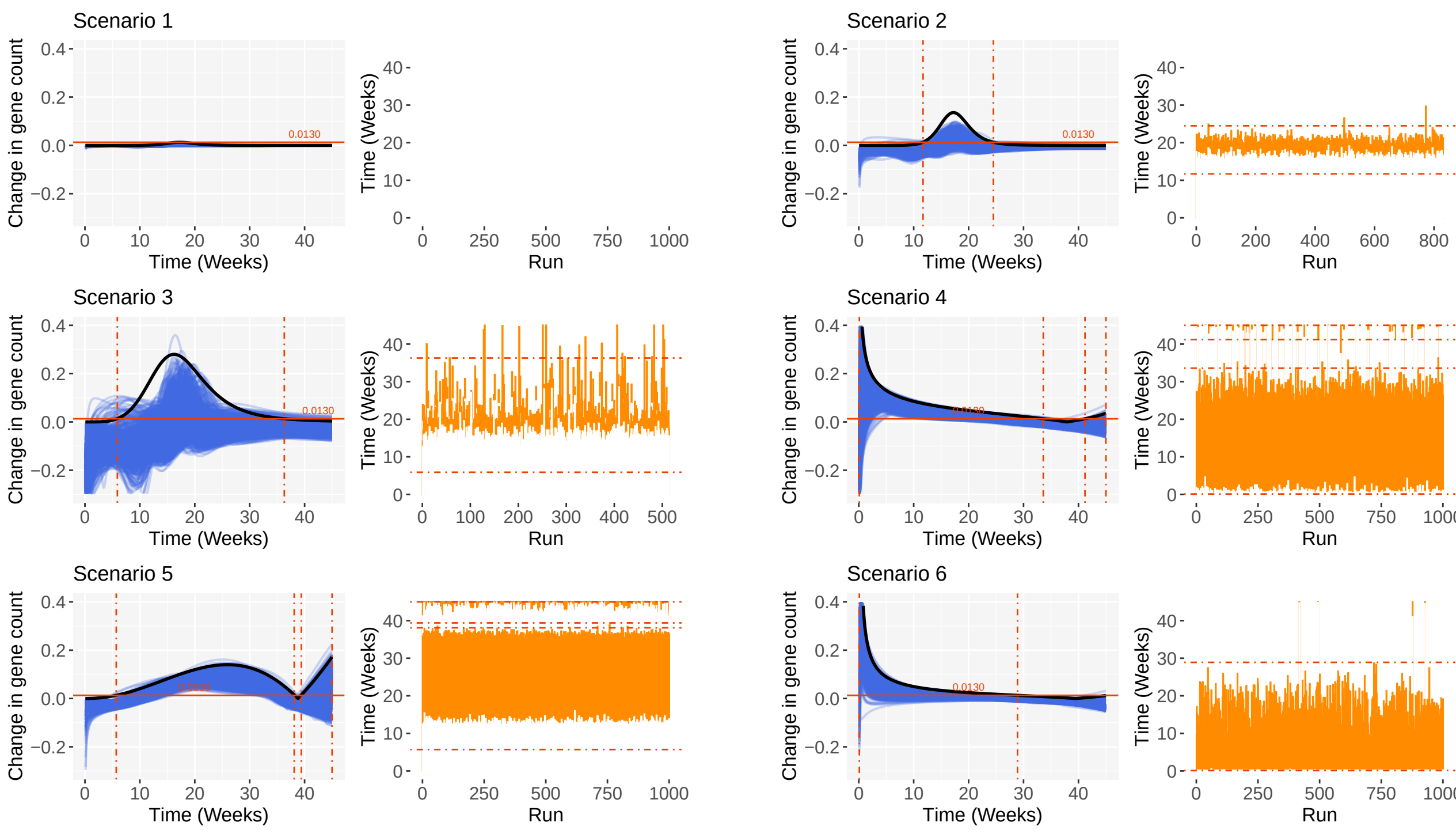

Figure S14: Results of the six simulation scenarios for a medium standard deviation. The left column depicts the true first derivative and the estimated confidence bands. The right column depicts the estimated time frames of significant change in gene expression for each run.

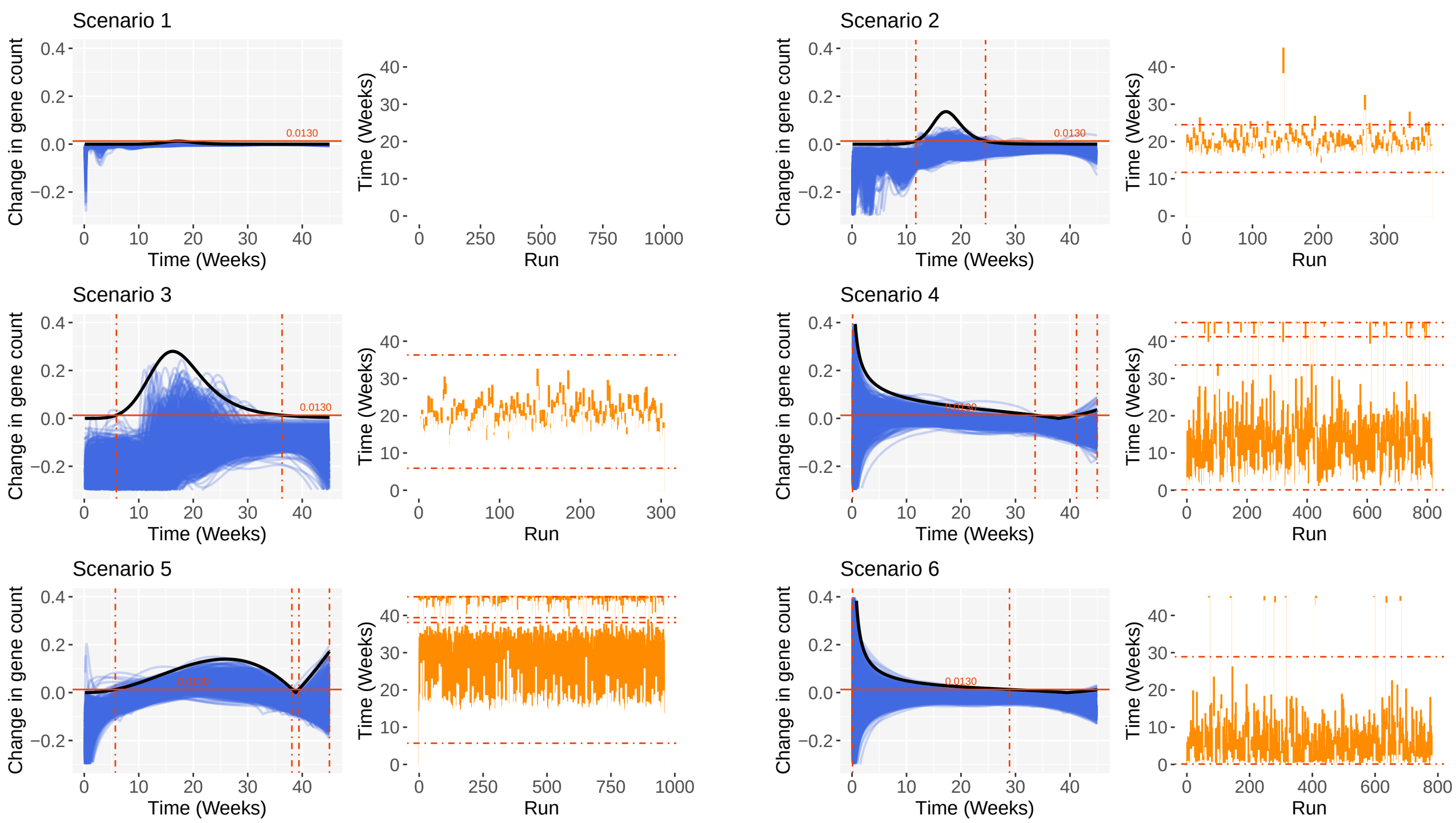

Figure S15: Results of the six simulation scenarios for a large standard deviation. The left column depicts the true first derivative and the estimated confidence bands. The right column depicts the estimated time frames of significant change in gene expression for each run.
